# Comparison of the differentiation of ovine fetal bone-marrow mesenchymal stem cells towards osteocytes on chitosan/alginate/CuO-NPs and chitosan/alginate/FeO-NPs scaffolds

**DOI:** 10.1038/s41598-023-50664-6

**Published:** 2024-01-02

**Authors:** Leila Soltani, Kambiz Varmira, Maryam Nazari

**Affiliations:** 1 Department of Animal Sciences, College of Agriculture and Natural Resources, Razi University, Kermanshah, 67144-14971 Iran; 2 Research Center of Oils and Fats, Kermanshah University of Medical Sciences, Kermanshah, Iran; 3 Applied Chemistry Department, Faculty of Chemistry, Razi University, Kermanshah, Iran

**Keywords:** Cell biology, Stem cells, Materials science

## Abstract

In the current study, the creation of a chitosan/alginate scaffold hydrogel with and without FeO-NPs or CuO-NPs was studied. From fetal ovine bone marrow mesenchymal stem cells (BM-MSCs) were isolated and cultivated. Their differentiation into osteocyte and adipose cells was investigated. Also, on the scaffolds, cytotoxicity and apoptosis were studied. To investigate the differentiation, treatment groups include: (1) BM-MSCs were plated in DMEM culture medium with high glucose containing 10% FBS and antibiotics (negative control); (2) BM-MSCs were plated in osteogenic differentiation medium (positive control); (3) positive control group + FeO-NPs, (4) positive control group + CuO-NPs; (5) BM-MSCs were plated in osteogenic differentiation medium on chitosan/alginate scaffold; (6) BM-MSCs were plated in osteogenic differentiation medium on chitosan/alginate/FeO-NPs scaffold; and (7) BM-MSCs were plated in osteogenic differentiation medium on chitosan/alginate/CuO-NPs scaffold. Alkaline phosphatase enzyme concentrations, mineralization rate using a calcium kit, and mineralization measurement by alizarin staining quantification were evaluated after 21 days of culture. In addition, qRT-PCR was used to assess the expression of the ALP, ColA, and Runx2 genes. When compared to other treatment groups, the addition of CuO-NPs in the chitosan/alginate hydrogel significantly increased the expression of the ColA and Runx2 genes (*p* < 0.05). However, there was no significant difference between the chitosan/alginate hydrogel groups containing FeO-NPs and CuO-NPs in the expression of the ALP gene. It appears that the addition of nanoparticles, in particular CuO-NPs, has made the chitosan/alginate scaffold more effective in supporting osteocyte differentiation.

## Introduction

According to Pittenger et al.^1^, mesenchymal stem cells (MSCs) can differentiate into fibroblasts, osteoblasts, chondrocytes, and adipocytes, among other cell types. They might be quickly proliferated ex vivo under the appropriate condition after being readily separated from bone marrow, adipose tissue, or peripheral blood^2^. Autologous MSCs are thus excellent candidates for use in tissue engineering and regenerative therapy^3–5^.

To ensure adequate bone healing in many skeletal problems like nonunion fractures, cervical and lumbar spine fusion, joint arthrodesis, and revision arthroplasty, more than 2.2 million bone grafting procedures (autologous bone graft and banked bone) are carried out annually throughout the world^6^. Unfortunately, the gold standard of bone grafting, autologous bone, needs to be removed from the patient during a separate surgery; surgery and recuperation durations lengthen as a result.

Potential issues include persistent discomfort at the donor site, infections, and eventual disability^7^. Tissue engineering provides a solution to get around those issues. According to the theory^8^, an extracellular matrix (ECM)-like structure can be formed by using a porous, biodegradable scaffold that encourages cell adhesion and proliferation. Previous research has demonstrated the significant potential for bone tissue engineering applications using natural-based polymers such as starch^9^ or chitosan^10,11^.

Low immunogenic potential, bioactive behavior, favorable interactions with host tissues, chemical adaptability, and high availability in nature are these materials' key benefits^7^.

Brown algae produce alginate, a linear anionic polysaccharide made up of repeating units of D-mannuronic acid and L-guluronic acid in various ratios. Due to its biocompatibility, low immunogenicity, water-retention capacity, and degradability, sodium alginate is a desirable material for wound dressing^12,13^.

Chitosan, a carbohydrate polymer made from the chitin of crustaceans, was selected for this study because of its biologically renewable, biodegradable, non-toxic, non-allergic, and bioactive mechanical qualities^14–18^. Based on the degree of chemical cross-linking, the chitosan monomer has variable biomechanical capabilities. However, the capacity of cells to adhere is compromised by this cross-linking process. The tissue engineering uses of pure chitosan continue to present difficulties. Chitosan has garnered a lot of interest when combined with other biomaterials that are enhanced with cell adhesion motifs.

Nanotechnologies have garnered significant attention in the field of bone tissue engineering, as they are associated with several disciplines such as physics, chemistry, engineering, biological sciences, and medicine. According to current definitions, nanomaterials are substances that have basic structural units that are smaller than 100 nm in at least one dimension and possess special qualities that set them apart from their bulkier counterparts and have helped them become very successful in a variety of biomedical applications^19–21^.

Nanoparticles have potential applications in drug/gene delivery, bio-imaging, cell labeling, pathologic diagnosis, and disease treatment^22–24^. They can also be used in tissue engineering scaffolds or surface coatings on implants. Numerous studies have been conducted to investigate the effects of nanoparticles on cell viability and functions, such as proliferation and differentiation, due to the interaction between nanoparticles and target cells^25,26^.

Bone repair requires stem cells that can differentiate into osteogenic cells, and FeO-NPs can provide the necessary proteins to regulate the proliferation and differentiation of these cells. Experiments with BM-MSCs and Fe-NPs coated with bovine serum albumin have shown an increase in the expression of collagenase-1 (Col1), alkaline phosphatase (ALP) activity, calcium deposition, and expression of osteocalcin (OC)^27^. Magnetic particles in combination with a pulsed magnetic field have a positive effect on osteogenic differentiation^28^.

Biopolymer scaffolds containing magnetic Fe-NPs significantly stimulate cell adhesion, osteogenic differentiation, and bone differentiation in vivo and in vitro, respectively. Increased gene expression of important transcription factors for osteoblasts (RUNX2 and Osterix) as well as the activity of ALP is evidence of this. Magnetic scaffolds are useful for bone repair, particularly when used with external magnetic stimulation as an adjuvant treatment^29^.

Trace elements such as iron, zinc, copper, and selenium play a vital role in bone metabolism^30^. The human body typically contains 50 to 120 mg of copper, with around two-thirds of the total copper found in muscles and the skeleton^31,32^. Copper is necessary for the formation of enzymes that help transfer electrons and reduce molecular oxygen, which is vital for cellular energy metabolism^33,34^. One of these enzymes, called lysyl oxidase, uses lysine and hydroxylysine as substrates to create cross-links required for the development of connective tissues, including bones^35,36^. Low concentrations of copper have been found to enhance the viability and growth of osteoblastic cells, which are responsible for bone formation. However, higher concentrations can be harmful and cause cytotoxicity^37^. Under normal physiological conditions, copper ions can create a hypoxic microenvironment similar to hypoxia-mimicking ions. This can increase bone mineral density by promoting the expression of bone-related genes such as ALP, OPN, and OPN. Copper achieves this by inhibiting active bone resorption and promoting angiogenesis, which is achieved through the up-regulation of vascular endothelial growth factor^38–40^. However, copper has been found to down-regulate the expression of Runx2 and other genes related to osteogenic differentiation. It also inhibits collagen formation while stimulating angiogenesis in vivo studies^41^. Studies have also suggested that copper interferes with mesenchymal stem cells involved in osteogenesis by affecting cytoskeleton organization, hindering cytoskeletal changes in BMSCs during osteogenic differentiation^41^. Copper can promote both osteogenesis and adipogenic differentiation of BMSCs, with a preference for the osteogenic lineage^42^. However, it is important to note that higher concentrations of copper can induce apoptosis in BMSCs^43^. Copper nanoparticles (Cu-NPs) have an antioxidant impact and are suitable for wound healing due to their biocompatibility and ability to stimulate the migration and proliferation of endothelial cells^44^. When used in scaffolds, copper ions can enhance bone tissue regeneration. In a study conducted by D'Mello et al.^45^, the effects of copper-doped chitosan scaffolds on bone regeneration were compared. The scaffolds were implanted in male Fisher 344 rats with 5 mm calvarial deficiencies. After 4 weeks, micro-CT scans and histological analysis revealed an increase in bone volume. The researchers concluded that the addition of copper ions to scaffolds may improve tissue regeneration^45^.

Lin et al.^46^ have developed a new type of copper-containing calcium phosphate cement (Cu-CPC) enriched with copper phosphate nanoparticles. The impact of these nanoparticles on osteogenesis and angiogenesis was studied on mouse BM-MSCs and human umbilical vein endothelial cells (HUVECs) at various concentrations. The results revealed that Cu-CPC with 0.01 weight percent and 0.05 weight percent of copper phosphate nanoparticles significantly increased BM-MSC adhesion and proliferation. The differentiation was supported by increased expression of genes related to osteogenesis (Runx2, Col-I, and OC) and angiogenesis (VEGF, eNOS, and FGF) and the creation of collagen type I. However, concentrations of copper phosphate nanoparticles greater than 0.05 weight percent reduced murine BM-MSC proliferation^46^.

Of importance, the incorporation of metal nanoparticles into hydrogels has been shown to enhance antimicrobial properties and accelerate osteogenic differentiation. Natural bone tissue consists of proteins and inorganic minerals^47^. Among different types of scaffolds, polymeric hydrogel scaffolds have gained remarkable interest because they are biocompatible, and the structures are similar to the macromolecular-based components in the body^48^.

To the best of our knowledge, the comparison of osteocyte differentiation of the chitosan-alginate scaffolds with or without CuO-NPs and FeO-NPs as a novel material has not been investigated yet. Therefore, this project aimed to fabricate lyophilized chitosan-alginate scaffolds contained with or without CuO-NPs and FeO-NPs and evaluate their effects on the proliferation, and osteocyte differentiation of ovine BM-MSCs. Also, using field emission scanning electron microscopy (FE-SEM) and tensile strength, respectively, the scaffolds' microstructure and compressive strength were examined. Using Fourier Transform Infrared (FT-IR) spectroscopy, the chemical bonds between chitosan, alginate, CuO-NPs, and FeO-NPs were studied. Furthermore, chitosan-alginate scaffolds with or without CuO-NPs and FeO-NPs, as well as their swelling and degradability were characterized. Moreover, Hydrogels were characterized by X-ray diffraction (XRD) and X-ray spectroscopy (EDS).

## Materials and methods

### Chemicals

Chemicals were obtained from Sigma–Aldrich chemicals if otherwise indicated.

### Preparation of the extract

Fruits of Maclura pomifera were purchased from a local herbal shop (Kermanshah, Iran). The fruits of Maclura pomifera were first cleaned with tap water and then with distilled water to remove any impurities before preparing the extract. The fruits were thinly sliced and dried for a week at room temperature in the shade. After drying, they were ground into a powder. 20 g of this powder was mixed with 200 mL of deionized water and heated at 80°C for 30 min using a heater stirrer. The solution was then centrifuged, and the supernatant was separated. Finally, the extract was filtered using Watman's paper and kept for future use.

### Preparation of iron oxide nanoparticles and copper oxide nanoparticles

FeCl_3_ was employed as the precursor salt and Maclura pomifera fruit extract as the natural reducing and capping/stabilizing agent in the synthesis of FeO-NPs. 50 mL of deionized water was used to make 10 mM of FeCl_3_. Salt and Maclura pomifera fruit aqueous extract were combined in a 1:1 volumetric ratio. By employing a 1 N NaOH solution, the pH was kept at 11.

The synthesis mixture was stirred at 80°C for 2 h. After cooling down to room temperature, the solution was centrifuged at 10,000 rpm. The resulting nanoparticles were washed multiple times with water and then dried at 50°C in an oven. Finally, to remove any remaining plant organic matter, the nanoparticles were calcined at 400°C in a muffle furnace, pulverized, and stored for further use.

Maclura pomifera fruit aqueous extract was used to produce copper oxide nanoparticles (CuO-NPs) by mixing it with a concentrated copper nitrate solution in a 1:1 ratio. The mixture was stirred at 80 °C for two hours, and the dark color of the solution indicated the synthesis of CuO-NPs. After incubating for 2 h, the colloidal was centrifuged at 10,000 rpm for 10 min to create a pellet. The pellet was cleaned three times with double-distilled water. To remove the associated plant organic matter, the final residue was calcined at 400 °C in a muffle furnace, pulverized, and kept for further use.

### Characterization of iron oxide and copper oxide nanoparticles

FE-SEM analytical techniques (LMU TESCAN BRNO-Mira3), the synthesized FeO-NPs and CuO-NPs were evaluated for their morphological characteristics. For the sensitive identification of chemical compounds, use FTIR (FTIR Bruker Alpha). It operates on the premise that practically every molecule in the electromagnetic spectrum can absorb light in the infrared range. The associated absorption peak that is produced identifies a specific molecular bond. The measurement's frequency range was between 4000 and 450 cm^-1^.

To understand the surface charge of CuO-NPs and FeO-NPs, the zeta potential (Horiba-SZ-100-Z model) was investigated at 25 °C. The chemical composition of the CuO-NPs and FeO-NPs was examined using energy-dispersive X-ray microanalysis (EDS, R Model Quan Tax 200, Germany). Additionally, to determine the size of the particles, FE-SEM pictures were also uploaded into Image J® 1.48 v software (National Institute of Health, USA).

### Preparation of chitosan/Alg/CuO-NPs or chitosan/Alg/FeO-NPs and chitosan/Alg scaffolds

**Figure 1:**
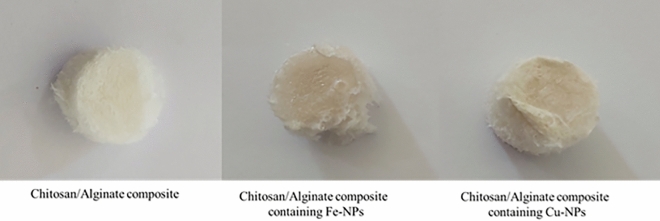
Different hydrogels after freeze-dry.

In 10 mL of deionized (DI) water, 0.1 mg each of CuO-NPs and FeO-NPs were added. To get a Cu or Fe nanoparticle solution, the mixture was agitated. To create a homogenous solution, 150 mg of low-molecular-weight chitosan powder was added to DI water containing each nanoparticle with 1% acid acetic, and 150 mg of alginate powder was put into the Cu or Fe nanoparticle solution under constant stirring to form a homogeneous solution. Each mixture was then poured into 48-well plates and put in the freezer for overnight at 80 °C. The mixture was then freeze-dried in a lyophilizer (VaCo 2-II, ZIRBUS) until dried, and the scaffolds were then cross-linked with 2% CaCl_2_ solution and washed with DI to remove unbound CaCl_2_. The scaffolds were kept at − 80 °C for 24 h and again freeze-dried at − 20 °C for 24 h. Chitosan/Alginate scaffolds were then created in the same way, but without the inclusion of CuO or FeO nanoparticles in the initial Chitosan/Alginate mixed solution (Fig. 1)^49^.

### In vitro FeO-NPs and CuO-NPs release

Atomic absorption Spectrophotometer (Varian Spectra AA 220 FSVarian) analysis was used to measure the quantity of iron and copper released from the chitosan/Alginate-CuO-NPs and chitosan/Alginate-FeO-NPs hydrogels. The tube was placed on a shaker and allowed to warm up. After 5cc of liquid free of the scaffold was removed from the tube, the liquid sample was examined to see how many FeO-NPs or CuO-NPs were present. Following collection, 5cc of Dulbecco's phosphate-buffered saline (DPBS) was added to the tube. The same process was carried out again at 1, 3, 5, 7, 12, and 24 h.

### Characterization of prepared scaffolds

#### Fourier-transformed infrared spectroscopy (FTIR)

The intermolecular interactions between the parts of the scaffolds were investigated using FTIR. The KBr disc method was used to record the spectra of chitosan, alginate, chitosan/alginate/CuO-NPs hydrogels, chitosan/alginate/FeO-NPs hydrogels, and chitosan/alginate/hydrogels in an FTIR spectrophotometer (FTIR Bruker Alpha) with a range of 400 to 4000 cm^-1^.

#### X-ray diffraction (XRD) study

An X-ray diffractometer (Intel: model Equniox 3000) was used to measure the X-ray diffraction patterns of the different hydrogels using Ni-filtered Cu radiation produced at 30 kV and 30 mA as the X-ray source. At a rate of 1° (2θ) per minute and a step of 0.1° (2θ), the diffraction patterns were calculated throughout a range of diffraction angles (2θ = 10° to 120°).

#### FE-SEM

By using a FE-SEM (LMU TESCAN BRNO-Mira3) with an acceleration voltage of 10 kV, the microstructure of the various hydrogels was examined. To improve conductivity before imaging, samples were sputter-coated with gold.

#### Swelling degree studies

Different hydrogels were studied for swelling in phosphate-buffered saline (PBS) solution (pH = 7.4) at room temperature. The equation below was used to calculate the equilibrium mass swelling of the hydrogel:

1 $$ {\text{Equilibrium}}\;{\text{mass}}\;{\text{swelling}} = {\text{m}}_{{1}} - {\text{m}}_{0} {\text{/m}}_{0} \times {1}00 $$

where m_0_ represents the weight of the dry hydrogel and m_1_ represents the weight of the hydrogel in its swelled state. A sample of each hydrogel was dried in a vacuum oven at 50 °C until the gel weight remained constant. The known amount of dry hydrogel of chitosan/alginate, chitosan/alginate containing CuO-NPs, and chitosan/alginate containing FeO-NPs was immersed in distilled water or PBS and kept at room temperature for seven days. After being taken out of the solution, the hydrogels were rapidly weighed and carefully cleaned using tissue paper. The hydrogel was weighed while it was swelling, and the equilibrium mass swelling % was determined using Eq. (1).

#### Weight loss analysis

The degradation rate of the produced hydrogels was calculated based on their weight loss. Each dried hydrogel was weighed and equal-weight samples were added to a falcon tube containing PBS at 37 °C. The weight loss was measured after 1, 3, 5, and 7 days. Triplicate samples were removed at the designated times and dried. The degree of degradation was calculated using Eq. (2), where W_0_ represents the initial weight of the hydrogels and W_1_ represents their dry weight after separation from water.

2 $$ {\text{Weight}}\;{\text{loss}}\;\% = {\text{W}}_{0} - {\text{W}}_{{1}} {\text{/W}}_{0} \times {1}00 $$

#### Tensile test

The mechanical properties of the hydrogels were evaluated using SANTAM STM-50 (ENG Design, Iran). Samples were prepared and subjected to tensile test at 25 ± 2 ºC with a crosshead speed of 2 mm/min.

#### Isolation and culture of ovine fetal BM-MSCs

Ovine fetuses (70–85 days old) were collected from the abattoir, maintained in DPBS, and brought to the lab on ice. The BM-MSCs were extracted from an ovine fetus by aspirating the femur and tibia with a solution of Dulbecco's modified eagle medium (DMEM) culture media supplemented with 100 IU/mL penicillin and 100 µg/mL streptomycin.

At room temperature, the bone marrow aspiration sample was centrifuged at a speed of 1900 g for 30 min on top of an equal amount of Ficoll. The cloudy layer was then gathered into a fresh tube and given two DPBS washes. DMEM media was supplemented with 5% fetal bovine serum (FBS), 2 mM L-glutamine, penicillin (100 IU/mL), and streptomycin (100 µg/mL) and 5 × 10^6^ cells/ml were cultivated in a T-25 flask. Cell number was counted using a hemocytometer. Cells were sub-cultured when they achieved 80% confluency and the media was replaced every three days^50^.

#### Induction of osteoblastic and adipogenic differentiation in ovine fetal mesenchymal stem cells

In 24-well plates, 1 × 10^4^ ovine fetal BM-MSCs were seeded in DMEM supplemented with 10% FBS, 1% penicillin–streptomycin, 10nM dexamethasone, 0.2mM sodium L-ascorbyl-2-phosphate, and 10mM-glycerol phosphate to stimulate osteoblastic differentiation. To stimulate adipogenic differentiation, another set of 1 × 10^4^ ovine fetal BM-MSCs were planted in 24-well plates for 21 days in DMEM enriched with 10% FBS, 1% penicillin–streptomycin, 10nM dexamethasone, and 5µg/ml insulin^51^.

Oil-red O staining was carried out for the evaluation of adipogenic differentiation. A 0.5% solution of Oil Red O was employed. Adipocytes were briefly fixed in 1% formaldehyde, washed in Oil Red O for 20 min, rinsed with 85% propylene glycol for 3 min, washed in distilled water, and mounted with an aqueous mounting solution. The osteogenic differentiated cells were washed in PBS, fixed in 4% paraformaldehyde, washed again in distilled water, incubated for 20 min at room temperature with an alizarin red solution (2% w/v alizarin red in DW, pH = 4.2), and then rinsed multiple times in distilled water^52^.

#### Cell proliferation

A 24-well tissue culture plate containing cell-seeded hydrogels was incubated in a medium for three days. The mitochondrial activity of ovine fetal BM-MSCs was evaluated using the MTT colorimetric test on the different hydrogels. This test helps in the identification of formazan crystals, which are formed from 3-(4,5-dimethylthiazolyl-2)-2,5-diphenyltetrazolium bromide. Cultures were treated with MTT (5 mg/mL) and incubated for 4 h, which led to the development of purple, insoluble formazan crystals due to the presence of living cells. The crystals were then dissolved using Dimethyl sulfoxide (DMSO), and the optical densities (OD) of the solutions were measured at 570 nm using a spectrophotometer^51^. The proportion of viable cells was estimated at 24 and 72 h by measuring the optical densities of the solutions.

$$ {\text{Viability}}\;\% = {\text{each}}\;{\text{sample`s}}\;{\text{ OD }}\;{\text{results/OD }}\;{\text{control}} \times 100. $$

#### Acridine orange/ethidium bromide (AO/EB) apoptosis assay

The technique of AO/EB staining was used to identify apoptotic cells. In a 24-well plate (4 × 10^5^ cells/well), ovine fetal BM-MSCs were cultivated and incubated for 72 h under various conditions. The cells were washed with DPBS, stained with 5 µL of the AO-EB working solution (100 µg/mL AO and 100 µg/mL EB in distilled water), and examined under a fluorescent microscope immediately^53^.

#### Induction of osteoblast differentiation of ovine fetal BM-MSCs

DMEM supplemented with 10% FBS, 100 IU/mL penicillin, 100 g/mL streptomycin, 10 nM dexamethasone, 0.2 mM sodium L-ascorbyl-2-phosphate, and 10 mM glycerol phosphate were used to stimulate osteogenic differentiation in ovine fetal BM-MSCs. Every third day, the differentiation medium was changed^54^.

To investigate the differentiation, treatment groups include: 1) ovine fetal BM-MSCs were plated in DMEM culture medium with high glucose containing 10% FBS and antibiotics (negative control); 2) ovine fetal BM-MSCs were plated in osteogenic differentiation medium (positive control); 3) positive control group + FeO-NPs, 4) positive control group + CuO-NPs; 5) ovine fetal BM-MSCs were plated in osteogenic differentiation medium on chitosan/alginate scaffold (hydrogel group); 6) ovine fetal BM-MSCs were plated in osteogenic differentiation medium on chitosan/alginate/FeO-NPs scaffold (hydrogel group containing FeO-NPs); and 7) ovine fetal BM-MSCs were plated in osteogenic differentiation medium on chitosan/alginate/CuO-NPs scaffold (hydrogel group containing CuO-NPs).

#### Alkaline phosphatase (ALP) activity and Ca content assay

The total protein of the cells was extracted using radio immune precipitation (RIPA) lysis buffer, followed by shaking for 4 h at 4 ºC, centrifuging for 15 min at 15,000 rpm at 4 ºC, and collecting the supernatant for the ALP activity assay on day 21 of cultured stem cells in osteogenic media. An ALP assay kit (PARS-AZMOON, Tehran, Iran) was used to assess the ALP activity. The activity was measured on a micro-plate reader at 405 nm (BioTek Instruments, USA).

The total quantity of protein (mg) was used to normalize the enzyme activity (IU/l). Using a calcium content test kit (PARSAZMOON, Tehran, Iran), the quantity of Ca^+2^ deposited on stem cells during osteogenic induction was quantified. Ca^+2^ was then removed from differentiated cells using 0.6 N HCl, and the mixture was shaken for 4 h at 4 ºC. Optical density (OD) was measured at 570 nm in a microplate reader (BioTek Instruments, USA) after the reagent was added to calcium solutions. The standard curve of OD versus a series of calcium concentration dilutions was used to determine the calcium content.

#### Alizarin red S staining

The cells were cleaned with PBS and then fixed in a 4% (v/v) paraformaldehyde solution. Next, Alizarin red S 2% (pH 4.2) was added to each well of a 24-well plate. The plates were then incubated at room temperature for 20 min and washed four times with dH2O, with each wash being shaken for 5 min^55^.

#### Quantification of mineralization

An earlier study^55^ was conducted to examine the calcium deposition in osteogenic media. Briefly, each treatment group received 300 µl of 10% (v/v) acetic acid. After 30 min, the monolayer was removed from the plate using a cell scraper and transferred to a 15-mL microcentrifuge tube containing 10% (v/v) acetic acid. The slurry was vortexed for 30 s, then 1.25 mL of mineral oil was added. The mixture was heated precisely to 85 °C for 10 min and then cooled on ice for 5 min. The slurry was then centrifuged for 15 min at 20,000 g, extracting 200 µL of the supernatant into a fresh 1.5-mL microcentrifuge tube. To neutralize the acid, 100 L of 10% (v/v) ammonium hydroxide was added. The supernatant was then tested using a 96-well format opaque-walled, transparent-bottomed plate, with triplicate 405 nm readings of aliquots (150 µL) performed.

#### Semi-quantitative-polymerase chain reaction (PCR)

The AccuZol (Bioneer) RNA extraction kit was used to extract total RNA. The RevertAid First Strand cDNA Synthesis Kit (Fermentas) was used to carry out reverse transcription. Table 1 shows the gene-specific primers used to assess the transcriptional activity of osteogenic markers: alkaline phosphatase (ALP), Runt-related transcription factor 2 (Runx2), and collagen type 1 (Col1A) by real-time PCR. Primers were created using the Oligo6 program.

**Table 1:** Lists the genes that were subjected to real-time PCR analysis, as well as the forward and reverse primers and product annealing temperatures.

Name of primer	Sequence (5'- > 3')	Length	Tm
Runx2			
	For: CCGCCGGACTCGAACTG	17	60
	Rev: GAGAGGCGCAGGTCTTGATG	20	
Col1A			
	For: CATGACCGAGACGTGTGGAA	20	60
	Rev: CATTCGTCCGTGGGGACTTT	20	
ALP			
	For: GGTACTTTGGGCGTAACAGCAG	22	60
	Rev: CGGAGAAGCATGAGTCACAGAG	22	
GAPDH			
	For: ATCGTGGAGGGACTTATGACC	21	60
	Rev: CGCCAGTAGAAGCAGGGATG	20	

Real-time PCR was performed in a 20 µl total volume using Fermentas' Maxima SYBR Green qPCR Master Mix kit. The PCR procedure consisted of 40 cycles of primary denaturation at 95 °C for 20 s, followed by annealing at 60 °C for 20 s, and elongation at 72 °C for 20 s. To standardize the level of expression for each of the examined markers, they were compared to the expression levels of the ovine housekeeping gene (GAPDH). The 2-CT technique was used to analyze the folds of the alterations after each reaction was run three times. The mean ± SD of the data, which were analyzed by SPSS using the ANOVA test, were reported. *p* < 0.05 was used to determine the threshold of significance.

### Statistical analysis

All data are shown as the mean ± standard derivation (SD). Statistical analysis was carried out using SPSS software. Statistical comparisons were performed using one-way analysis of variance (ANOVA) with Duncan’s test. Differences were considered statistically significant at *p* < 0.05.

### Ethics statements

All the methods were carried out in accordance with relevant guidelines and regulations. This study was approved in advance by Ethics Committee of Kermanshah University of Medical Sciences (ethic number: IR.KUMS.REC.1400.403).

## Results

**Figure 2:**
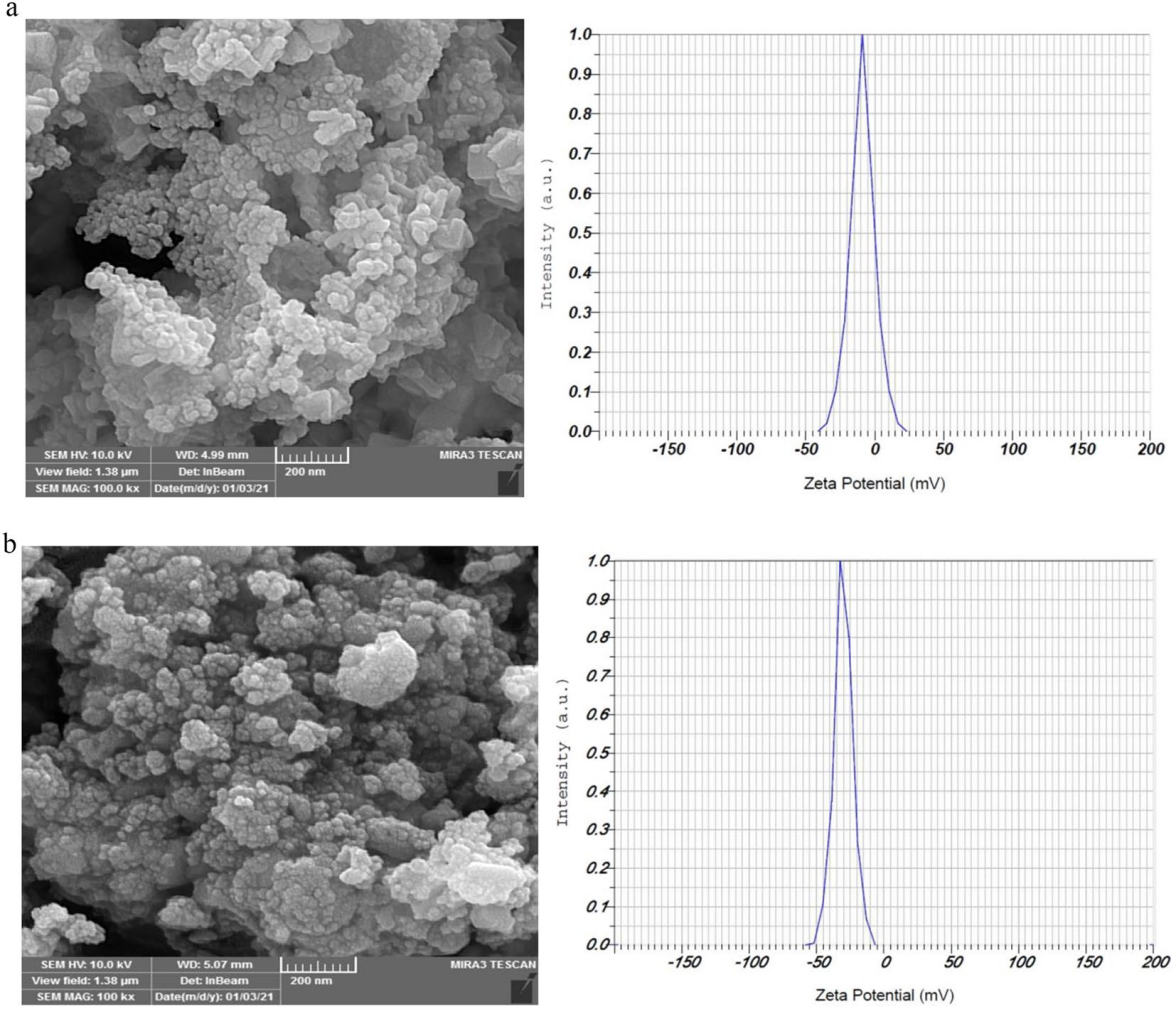
FE-SEM images and Zeta-potential of (**a**) FeO-NPs and (**b**) CuO-NPs.

Following evaluation in Image J software, FE-SEM pictures of FeO-NPs and CuO-NPs synthesized from Maclura pomifera fruit extract also demonstrate their sizes, which are 105.45 and 56.65 nm, respectively. To examine the stability of nanoparticles, zeta potential analysis was used, and the results are depicted in Fig. 2. The stability of the nanoparticles is attributed to the associated phytonutrients on their surface, as indicated by the zeta potentials of the synthesized FeO and CuO green nanoparticles of − 7.9 mV and − 30 mV, respectively.

After green synthesis, the CuO-NPs were examined using an EDS energy dispersive spectrometer to identify the types of elements present. Suppl. 1 depicts the EDS spectrum for CuO-NPs produced by Maclura pomifera fruit extract. This spectrum contains 44.69%, 10.28%, 5.91%, and 38.98% of copper, carbon, nitrogen, and oxygen components, respectively (Supplementary Fig. 1a).

EDS analysis of Green FeO-NPs confirms the presence of the Fe element (Supplementary Fig. 1b). The spectrum also reveals the existence of other elements, including oxygen, nitrogen, and carbon. The presence of non-metallic components other than iron indicates that the organic chemicals in the fruit extract from American Maclura pomifera successfully transformed iron ions into nanoparticles. Furthermore, the high signal of elemental iron in the EDS spectrum proves the existence of elemental iron in the sample.

The peaks observed in the range of 3552, 3476, and 3414 cm^-1^ wave numbers correspond to the OH group present in the structure of potential macromolecules that act as a stabilizing or reducing agent of copper nitrate to CuO-NPs, as demonstrated by the results of the FTIR analysis.

**Figure 3:**
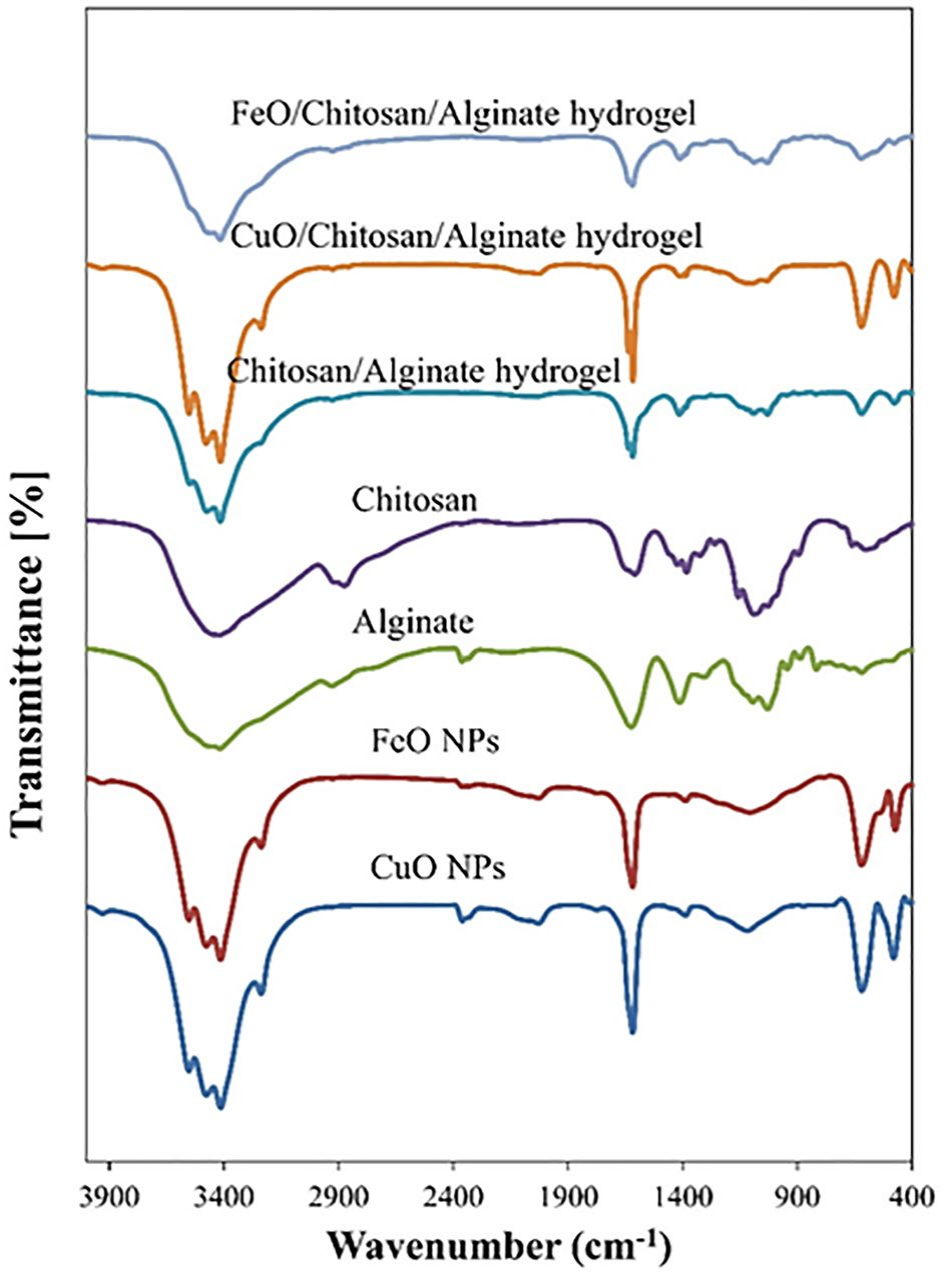
FT-IR spectrum of the CuO-NPs; FeO-NPs; alginate; chitosan; chitosan/alginate hydrogel; chitosan/alginate contain FeO-NPs and chitosan/alginate contain CuO-NPs.

Additionally, the 1388 cm^-1^ wave number has peaks that are associated with the CH_3_ bending group, N–O, or C-N related to organic chemicals that are present in CuO-NPs as a masking factor during nanoparticle formation. It displays bands at 2360, 1618, and 1118 cm^-1^, which correspond to the stretching of the C-O molecule and the hydroxyl group (-OH). The absorption of CuO is related to the 620 and 481 cm^-1^ bands (Fig. 3 and Supplementary Table 1).

The presence of OH functional groups, aliphatic and aromatic H-C, as well as carbonyl groups, can be seen in the infrared absorption spectrum of FeO-NPs synthesized by American Maclura pomifera extract in Fig. 3. This is consistent with the secondary compounds present in American Maclura pomifera fruit extract. Strong absorption bands were visible in these spectra at 1118, 1388, 1618, and 3414 cm^-1^. The stretching vibrations of methylene, carbonyl groups, aromatic stretching, H-C stretching, and C-O stretching are represented by these bands. The IR-FT spectrum supports the contribution of these functional groups to the biotransformation of American Maclura pomifera fruit extract to the production and stability of FeO-NPs. The Fe–O stretching band vibration is responsible for the absorption peak in the FT-IR spectra at around 620 cm^-1^ (Fig. 3 and Supplementary Table 1).

Figure 3 and Suppl 1 infrared spectrum of alginate revealed a peak at 3454 cm^-1^, which represented the –OH group of carboxyl, as well as peaks at 1622 cm^-1^ and 1411 cm^-1^, which represented the asymmetric and symmetric vibration of –COOH groups, and a strong peak at 1021 cm^-1^, which represented the C-O vibration of carboxylic acid. Chitosan was found to have N–H peaks at 1615 cm^-1^ and 3434 cm^-1^ (overlapping O–H and NH_2_ vibration), 1081 cm^-1^ (C-N stretching vibration), and 1329–1426 cm^-1^ (C–OH deformation vibration). A decrease in absorption intensity at 3414 cm^-1^ (-OH and NH_2_ vibration), which is brought on by the protonation of the COOH group to COO^−^ and NH_2_ to NH_3_^+^ on the ionic interaction between the carbonyl group of alginate and the amino group of chitosan, was used to demonstrate the formation of alginate-chitosan hydrogel as a polyelectrolyte. According to Suratman et al.^56^ and Li et al.^57^, the complex between alginate and chitosan occurred as evidenced by the change of the peak from 1615 to 1622 cm^-1^.

The characteristic for carbonyl groups (-COOH and CO) in chitosan/alginate hydrogel containing CuO-NPs and FeO-NPs was changed from 1622 to 1620 cm^-1^ and from 1411 to 1386 cm^-1^ for the scaffold containing CuO-NPs and 1402 cm^-1^ for the scaffold containing FeO-NPs, respectively, when compared to chitosan/alginate scaffolds. In contrast, the peak of the hydroxyl group (-OH) was similarly moved and intensified from 3420 to 3414 cm^-1^ when comparing the chitosan/alginate scaffold with chitosan/alginate/CuO-NPs and chitosan/alginate/FeO-NPs. Cu–O bond-related peaks for CuO-NPs may be detected at 611 and 481 cm^-1^ and 620 and 471 cm^-1^ for chitosan/alginate/CuO-NPs, respectively^58,59^. Additionally, according to Rada et al.^60^, the peak associated with the Fe–O bond in FeO-NPs can be detected at 601 cm^-1^, while the peak associated with the same bond for chitosan/alginate/FeO-NPs can be seen at 621 cm^-1^.

**Figure 4:**
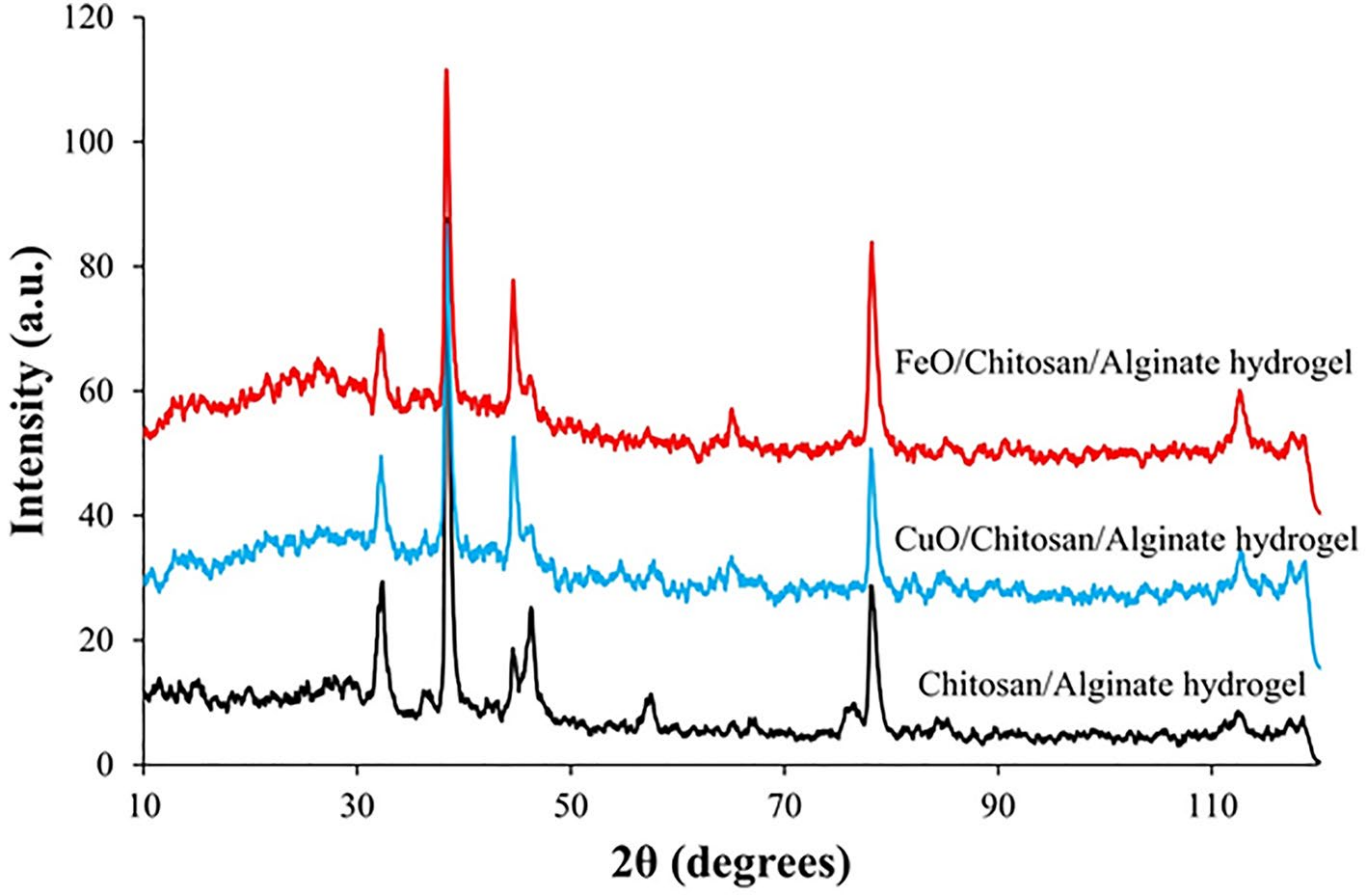
XRD patterns of the chitosan/alginate hydrogel; chitosan/alginate contain FeO-NPs and chitosan/alginate contain CuO-NPs.

The cross-linking of chitosan-alginate hydrogel by calcium chloride results in the crystalline nature of the synthesized chitosan-alginate hydrogel, according to XRD data. Additionally, the crystal structure of chitosan-alginate hydrogels containing Cu-NPs and Fe-ONPs can be seen clearly from their XRD patterns. For the XRD peaks in the 2θ range (10˚ < 2θ < 30˚), the somewhat amorphous character was attributed to the amorphous feature brought on by alginate and chitosan. Strong ionic interactions between the parts were responsible for a small change in the scaffold's 2θ values^61^. All Cu-NPs and FeO-NPs had diffraction patterns that were comparable to XRD peaks in the 2θ range (30˚ < 2θ < 80˚). CuO nanoparticles were found to be crystalline as evidenced by sharp and strong peaks at 32.5°, 39.2°, 46.5°, and 78.5° and weak peaks at 36.3°, 54°, and 58°. The crystalline CuO nanoparticles are present in the chitosan-alginate hydrogel containing Cu-NPs as shown by the strong peaks seen at ~ 32°, 39° and 45° (2θ values). Also, strong peaks observed at ~ 32°, 38°, 44° and 65° and weak peaks at ~ 30° and 76° indicate the presence of crystalline FeO-NPs in chitosan-alginate hydrogel containing FeO-NPs (Fig. 4)^59,62–68^.

**Figure 5:**
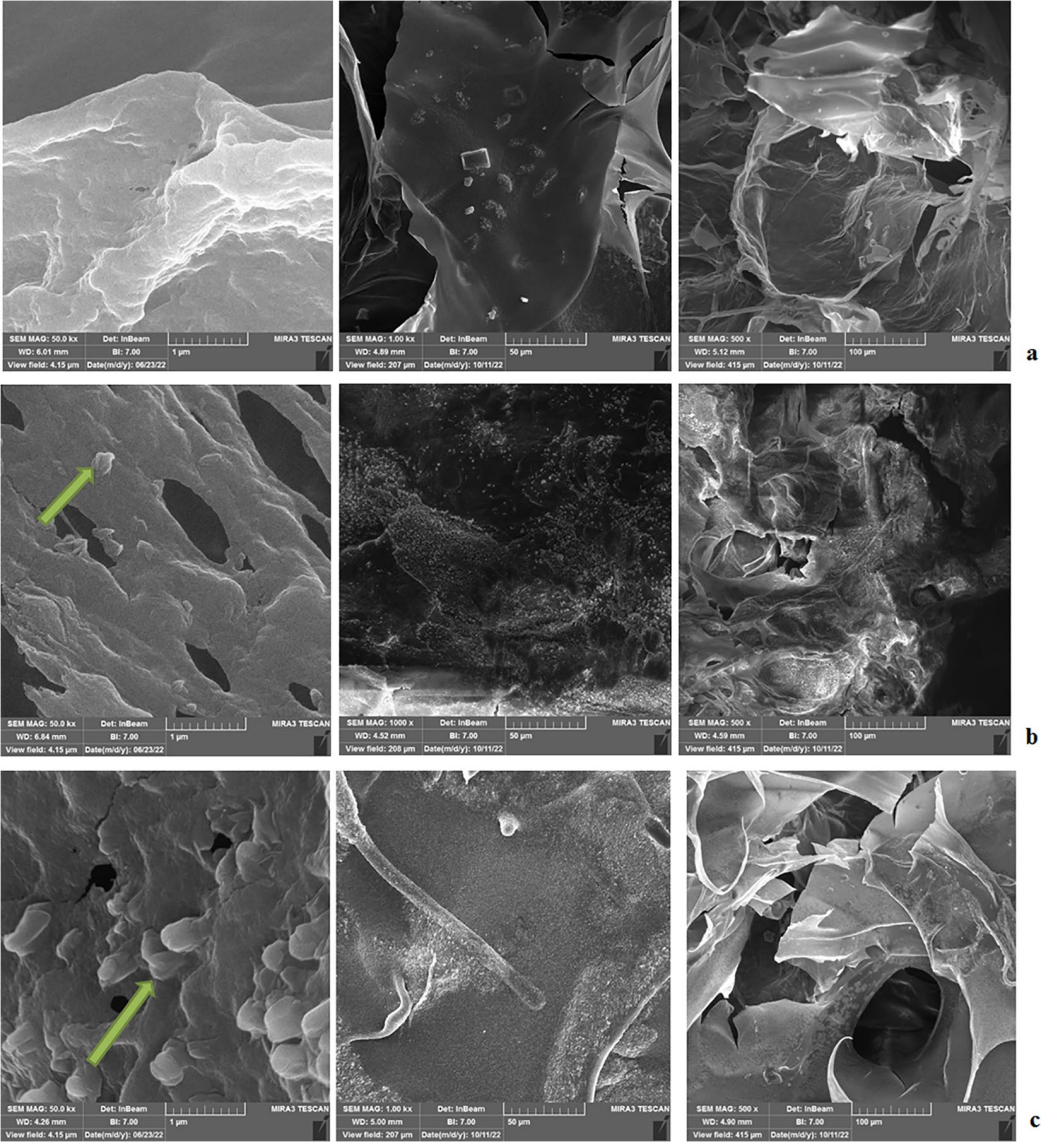
FE-SEM image of (**a**) chitosan/alginate hydrogel; (**b**) chitosan/alginate hydrogel contain FeO-NPs; (**c**) chitosan/alginate hydrogel contain CuO-NPs. The arrows refer to the nanoparticles inside the hydrogels.

The FE-SEM findings of several chitosan/alginate scaffolds with and without CuO-NPs and FeO-NPs are shown in Fig. 5. The raised bumps coated in chitosan/alginate hydrogel are visible at the smallest size (1 µm). These spots are not visible in the group of chitosan/alginate hydrogel without nanoparticles, which may be a sign that nanoparticles are present in the scaffold. These elevated patches are also seen at a scale of 50 µm. A greater scale of 100 µm has revealed the presence of porosity in the scaffolds, akin to the extracellular matrix in that it can serve as a site for cell attachment and implantation.

**Figure 6:**
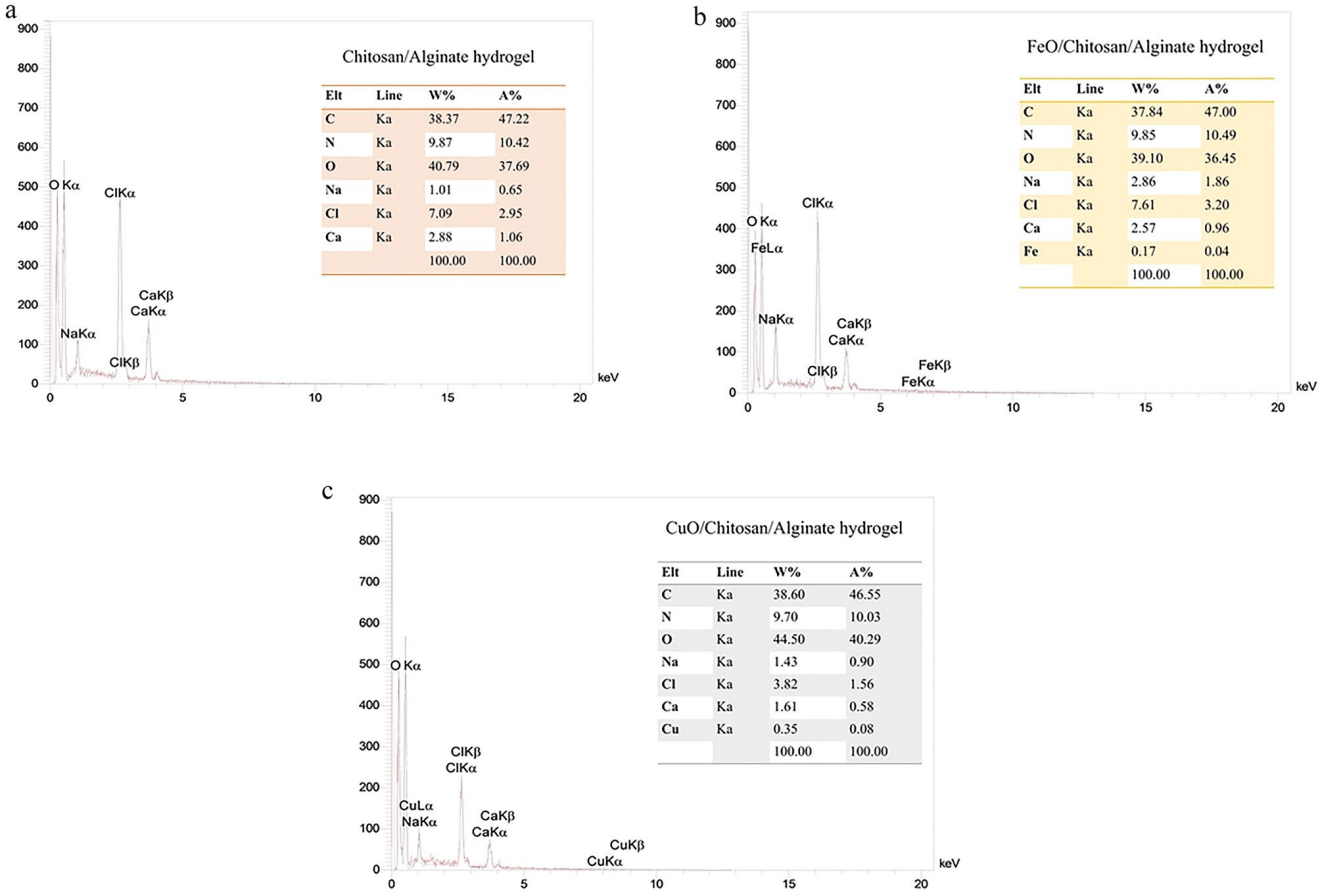
EDS image; (**a**) chitosan/alginate hydrogel; (**b**) chitosan/alginate containing FeO-NPs; (**c**) Chitosan/alginate composite containing CuO-NPs.

Figure 6 displays the findings of an EDS investigation of chitosan/alginate hydrogels with or without FeO-NPs or CuO-NPs, respectively. According to EDS analysis, chitosan/alginate hydrogel contains carbon, nitrogen, oxygen, sodium, calcium, and chlorine (Fig. 6a). Chitosan/alginate containing FeO-NPs showed carbon, nitrogen, oxygen, sodium, calcium, chlorine, and iron (Fig. 6b). Similarly, chitosan/alginate hydrogel containing CuO-NPs showed carbon, nitrogen, oxygen, sodium, calcium, chlorine, and copper (Fig. 6c).

**Figure 7:**
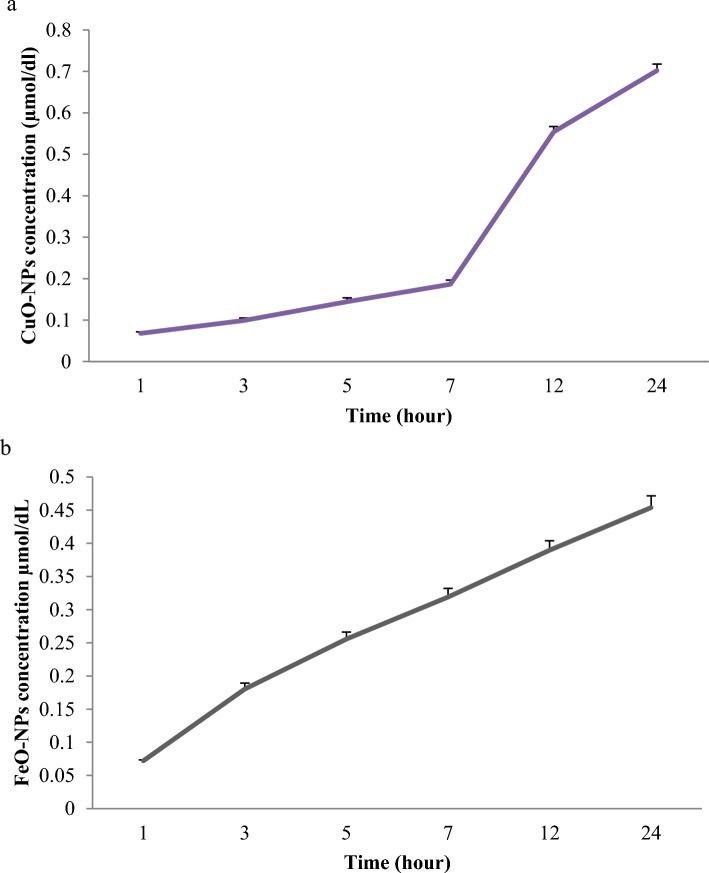
Release of CuO-NPs (**a**) from the Chitosan/alginate composite containing CuO-NPs and FeO-NPs (**b**) from the Chitosan/alginate composite containing FeO-NPs immersed in PBS.

Figure 7 displays the findings of the release of CuO-NPs and FeO-NPs loaded in the chitosan/alginate hydrogels at various points following the atomic absorption spectrophotometer assessment throughout 24 h. The release of FeO-NPs displays an increased tendency at various points in time (Fig. 7a). For the CuO-NPs, the release process was slow at 1, 3, 5, and 7 h, but after that, an increase in the release rate was seen (Fig. 7b).

**Figure 8:**
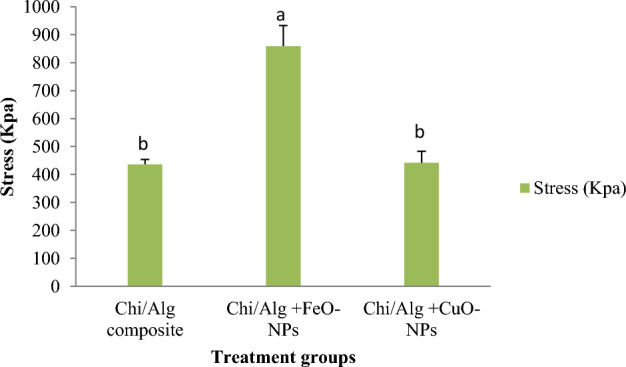
Compressive strength of synthesized various hydrogels, mean ± SD, n = 3, *p* < 0.05.

The compressive strength of several hydrogels was assessed in the current study. The resistivity of the chitosan/alginate hydrogel containing FeO-NPs was significantly higher than that of the chitosan/alginate hydrogel and the hydrogel containing CuO-NPs (*p* < 0.05; Fig. 8). However, there was no significant change between two chitosan/alginate hydrogel and chitosan/alginate hydrogel containing CuO-NPs (*p* > 0.05).

**Figure 9:**
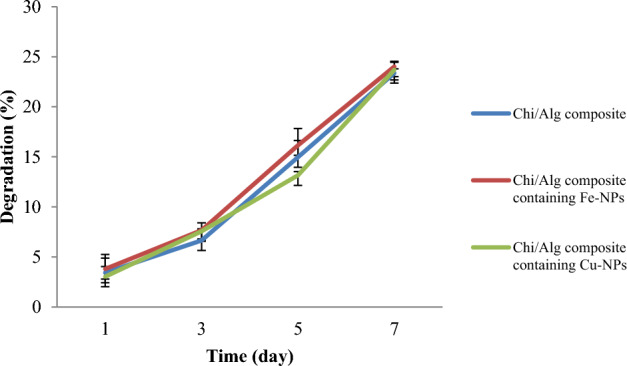
Degradation studies of chitosan-alginate hydrogel, chitosan-alginate/CuO-NPs hydrogel and chitosan-alginate/FeO-NPs hydrogel.

For application in biomedicine, hydrogel degradation must occur at the proper rate. Figure 9 illustrates the residual weight of several hydrogels after seven days. During this time, there was no significant difference in the rate of degradation between chitosan/alginate hydrogels, chitosan/alginate hydrogels containing FeO-NPs, and chitosan/alginate hydrogels containing CuO-NPs. All of these hydrogels displayed essentially the same pattern of degradation.

Table 2 details the findings regarding the level of moisture absorption in various scaffolds (chitosan/alginate, chitosan alginate containing FeO-NPs, or chitosan alginate containing CuO-NPs) during a 7-day incubation period. All hydrogels exhibited an increase in moisture absorption on various days, but on days 1, 3, and 5, the chitosan/alginate hydrogel showed a significantly higher percentage of water absorption than the hydrogels containing FeO-NPs or CuO-NPs (*p* < 0.05). On day 7, there was no significant difference between the chitosan/alginate hydrogel containing FeO-NPs and the chitosan/alginate hydrogel containing CuO-NPs (*p* > 0.05). However, there was a significant difference in the amount of moisture absorption between these two hydrogels compared to the chitosan/alginate hydrogel (*p* < 0.05). During the swelling process, the intermolecular spaces and volume expanded due to interactions between various groups and water molecules.

**Table 2:** Swelling behavior of the scaffolds immersed in PBS.

Time	Water absorption (%)		
	Chitosan/alginate composite	Chitosan/alginate composite contain FeO-NPs	Chitosan/alginate composite contain CuO-NPs
24h	1071 ± 25.47^a^	966 ± 11.04^b^	989 ± 2.18^b^
72h	1145 ± 44.10^a^	880 ± 41.37^b^	921 ± 43.78^b^
5 days	1099 ± 61.51^a^	710.72 ± 6.44^c^	789 ± 18.17^b^
7 days	930 ± 21.91^a^	749 ± 69.34^b^	871 ± 10.50^b^

Different superscripts within the same row demonstrate significant differences (*p* < 0.05), whereas the same superscripts do not demonstrate significant differences (*p* > 0.05).

**Figure 10:**
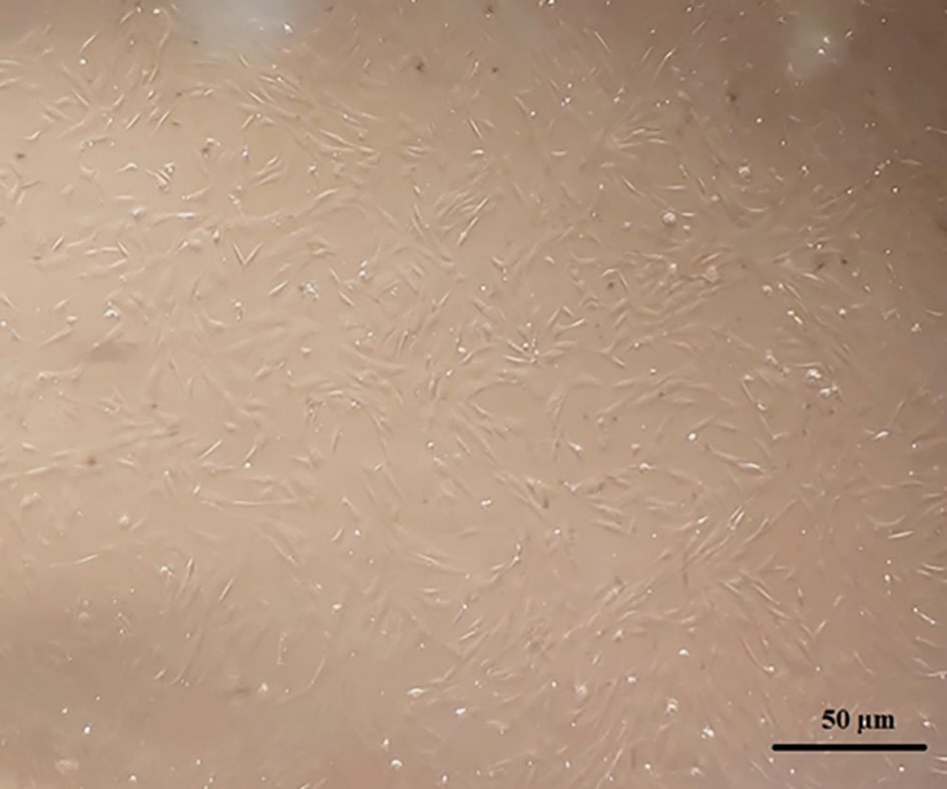
Ovine fetal BM-MSCs of the second passage.

**Figure 11:**
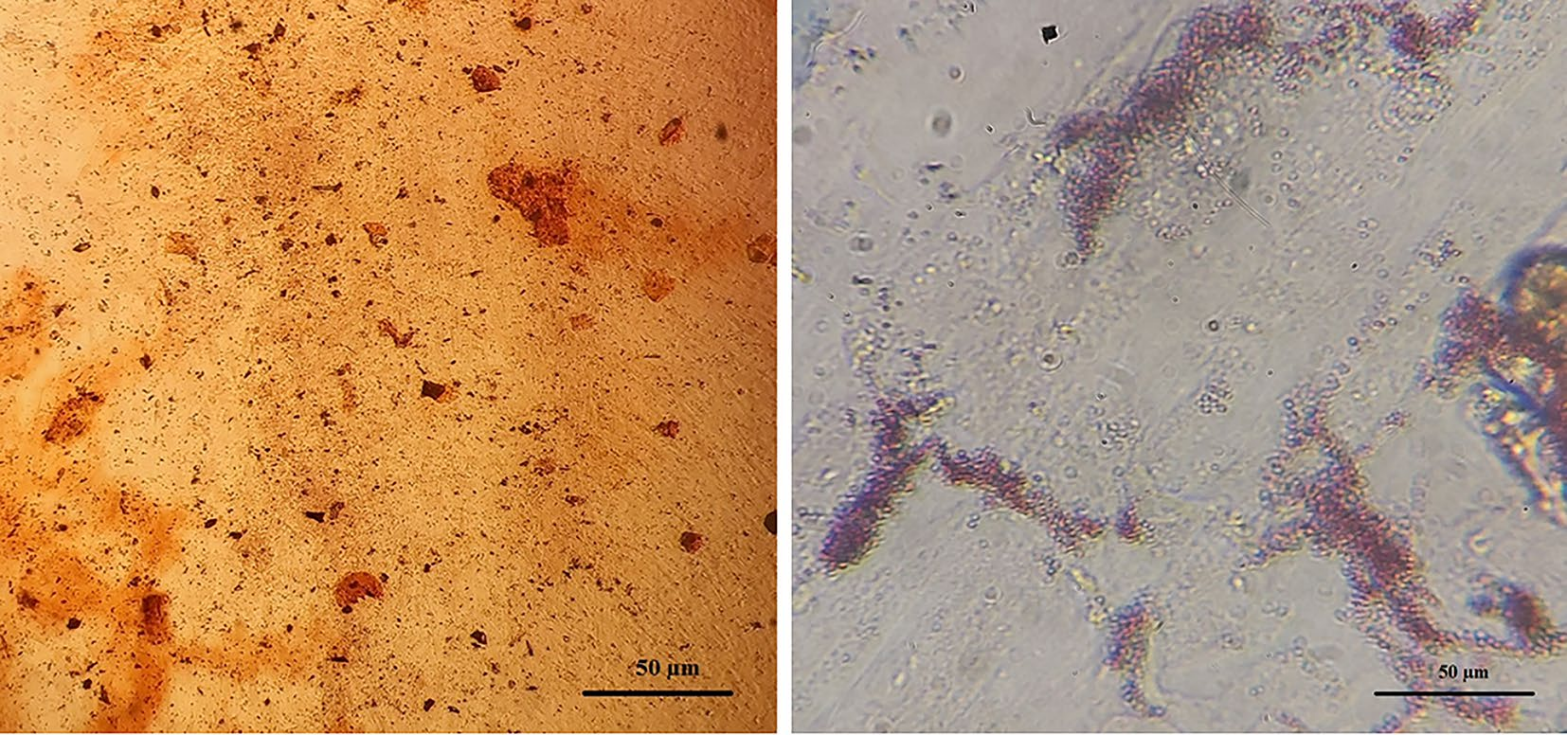
Differentiation into adipose cells (right) and octocyte cells (left) of ovine fetal BM-MSCs.

After 48 h from the start of the initial culture, fibroblast-like cells that attached to the bottom of the culture flask were evident under the microscope. These cells had spindle- or triangle-shaped cell bodies, a big, oval nucleus, and a cell colony that was expanding outward. These cells rapidly proliferated during the initial culture and reached confluence after five days (Fig. 10). Mesenchymal stem cells can differentiate into mesodermal cell lines such as osteocytes, adipose, and chondrocyte cells. To confirm the stemness of the isolated cells, osteogenic and adipogenic differentiation mediums were used. At the end of the 21-day osteogenic or adipogenic differentiation period, oil red staining was used to confirm the differentiation into adipocytes, and alizarin was performed to confirm differentiation into osteocytes. The spindle-shaped ovine fetal BM-MSCs started to become cubic in the osteogenic differentiation media, and the mineralized foci showed up under the microscope as red-speckled points after being stained with alizarin red. After oil red staining, the morphological alterations associated with adipogenic differentiation could be seen as the emergence of vacuoles holding fat droplets (Fig. 11).

**Figure 12:**
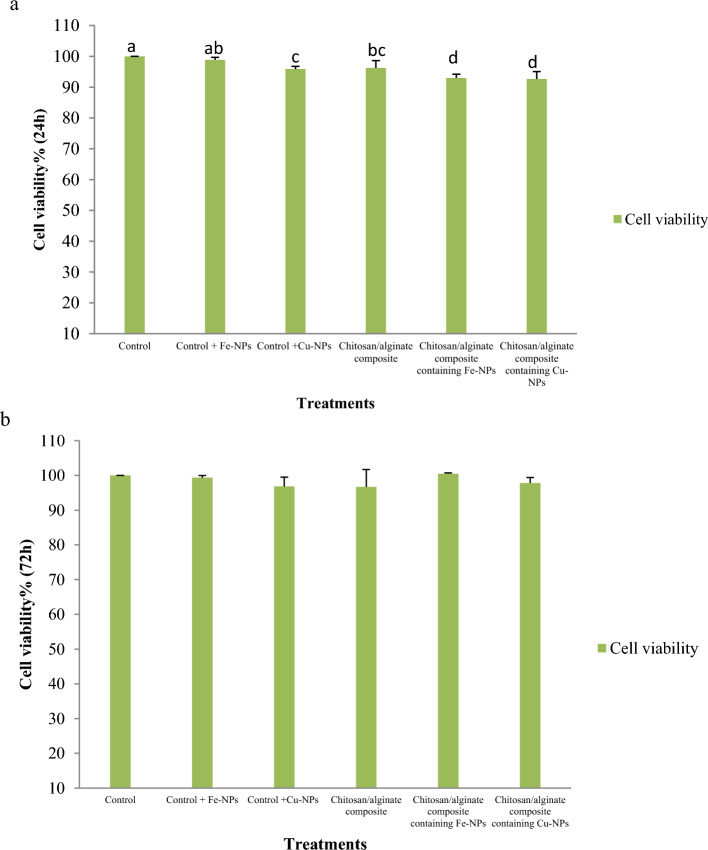
Cell viability of ovine fetal BM-MSCs in various treatment groups. (**a**) MTT test after 24 h of culture (*p* < 0.05); (**b**) MTT assay after 72 h of culture.

**Figure 13:**
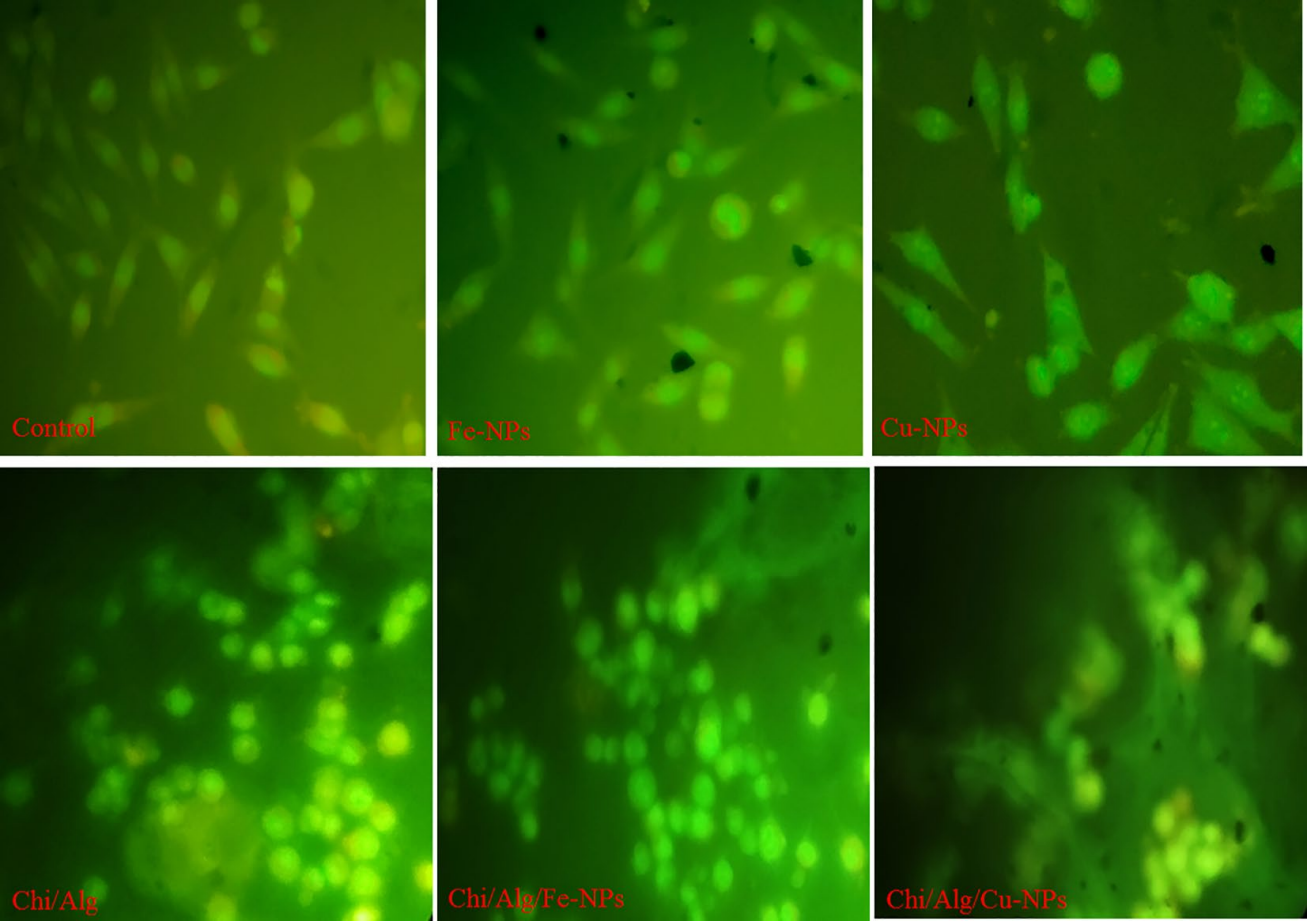
Cells cultivated with various treatment groups were stained fluorescently with Acridine orange-ethidium bromide (AO/EB) to identify apoptotic morphology.

Ovine fetal BM-MSC viability rates decreased after 24 h on scaffolds with or without CuO-NPs and FeO-NPs (Fig. 12a; *p* < 0.05), however after 72 h of incubation on scaffolds with or without CuO-NPs and FeO-NPs, there was no significant difference in viability rates (*p* > 0.05; Fig. 12b). To validate the cytotoxicity assay, cells in the different treatment groups were stained with acridine orange/ethidium bromide to assess the degree of cell apoptosis. After 72 h of cell proliferation, the findings of the apoptosis assay revealed a decline in cytotoxicity in various treatment groups. The cell nuclei were shown in green in each treatment group (Fig. 13).

**Figure 14:**
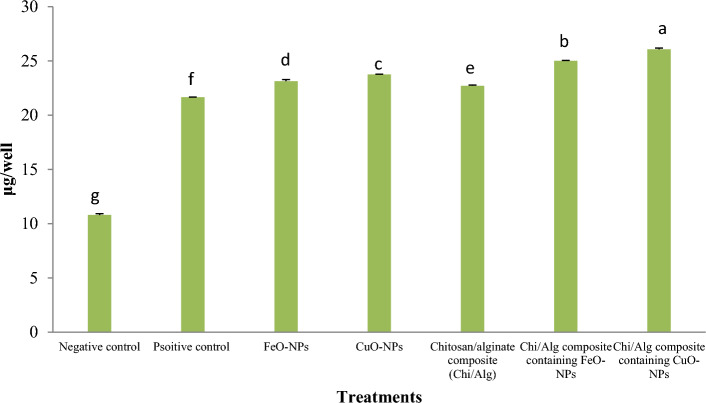
Calcium values from scaffolds at day 21. (mean ± SD; n = 3 biological replicates; *p* < 0.05). Different English letters on distinct columns reflect variations between treatment groups that are significantly different from one another.

After BM-MSCs were cultured for 21 days in osteogenic culture media with the treatment groups identified in this study present, the calcium deposition assay was conducted; the findings are displayed in Fig. 14. The inclusion of CuO-NPs in the chitosan/alginate hydrogel significantly improved the differentiation of BM-MSCs into osteocytes as compared to other treatment groups (*p* < 0.05). FeO-NPs in chitosan/alginate hydrogel significantly enhanced (*p* < 0.05) calcium deposition compared to other treatment groups, including chitosan/alginate hydrogel, groups containing CuO-NPs or FeO-NPs, positive control, and negative control. The addition of CuO-NPs into osteogenic media significantly increased calcium deposition as compared to the chitosan/alginate hydrogel, the group containing FeO-NPs, and the positive and negative control groups (*p* < 0.05, Fig. 14).

**Figure 15:**
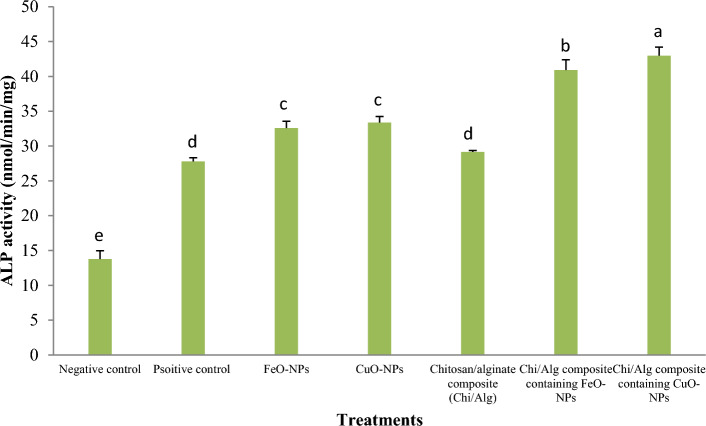
Each group's cells were cultivated, and on day 21 the ALP activity was assessed. (mean ± SD; *p* < 0.05; n = 3 biological replicates). Different English letters on distinct columns show significant differences between treatment groups.

Figure 15 displays the results of alkaline phosphatase enzyme activity levels observed after 21 days of ovine fetal BM-MSC culture in osteogenic media among different treatment groups. The use of chitosan/alginate scaffold containing CuO-NPs significantly improved the alkaline phosphatase enzyme activity (*p* < 0.05) compared to other groups. Except for the hydrogel containing CuO-NPs, the use of FeO-NPs in the chitosan/alginate hydrogel significantly increased the quantity of alkaline phosphatase (*p* < 0.05) compared to the other groups. Moreover, the addition of FeO or CuO nanoparticles in the osteogenic culture media of ovine fetal BM-MSCs significantly increased the rate at which alkaline phosphatase enzyme was produced (*p* < 0.05) compared to the chitosan/alginate hydrogel, the negative and positive control groups.

**Figure 16:**
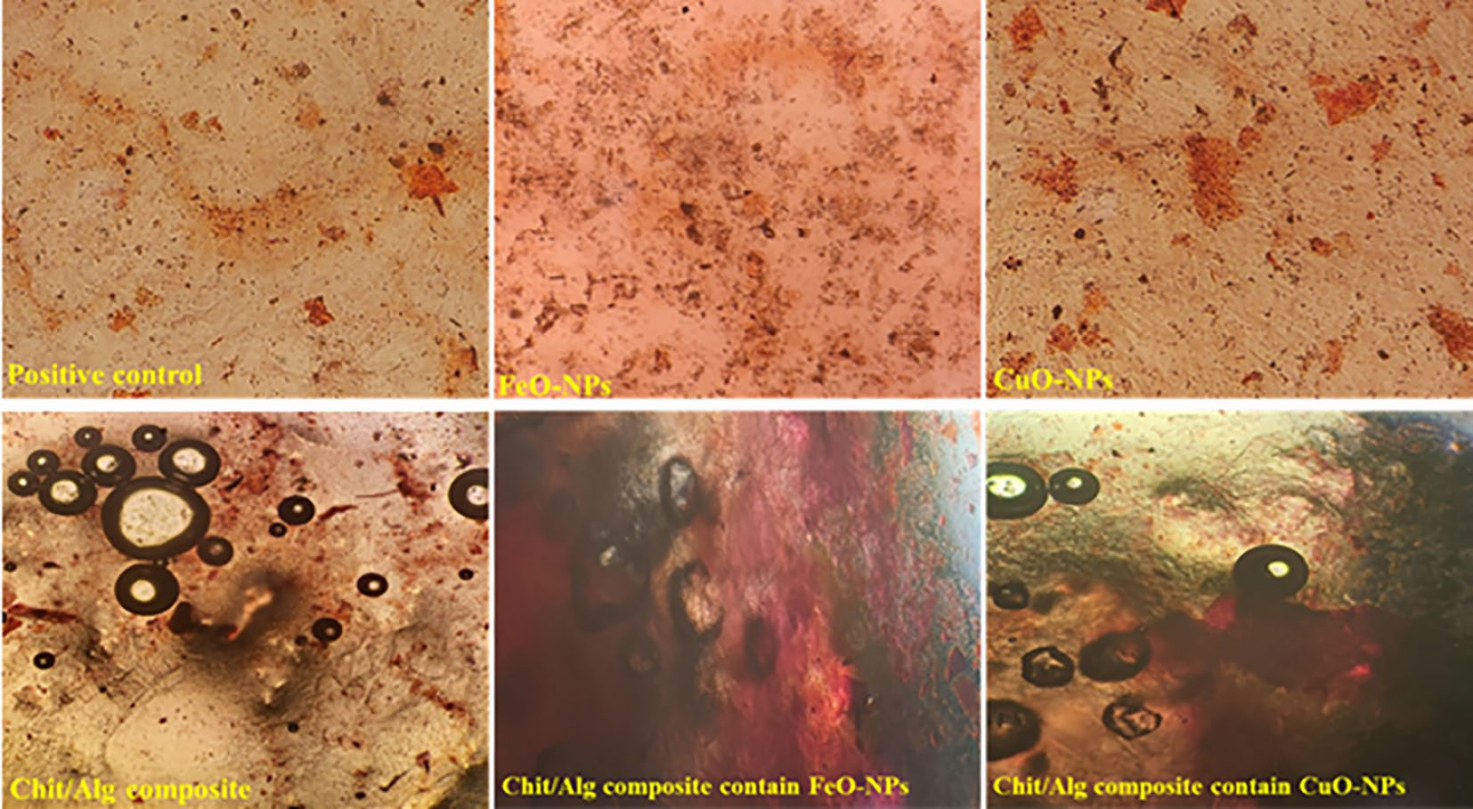
Inverted light microscopic image presenting Alizarin red S staining for different ovine fetal BM-MSCs experimental groups.

**Figure 17:**
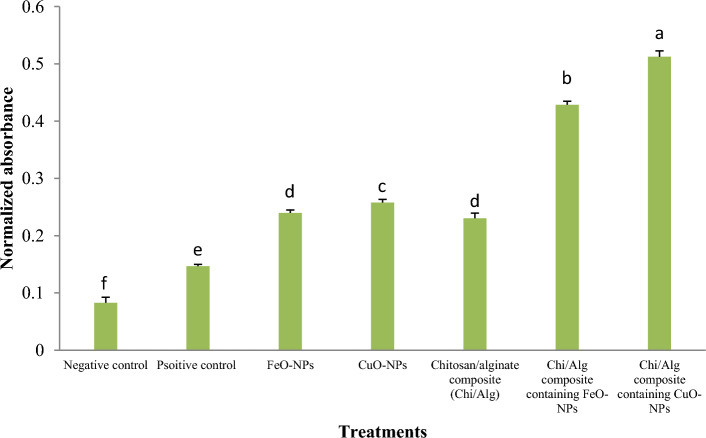
Analysis of the optical density at 405 nm of the alizarin red staining after 21 days (n = 3 biological replicates; mean ± SD; *p* < 0.05). Different English letters on distinct columns reflect variations between treatment groups that are significantly different from one another.

The results of the alizarin red staining demonstrated that hydroxyapatite deposits were present in all treatment groups (Fig. 16). Among the treatment groups, the chitosan/alginate containing CuO-NPs displayed the highest amount of calcium deposits. This finding was statistically significant when compared to the other treatment groups, as confirmed by quantifying the alizarin red staining findings (Fig. 17).

**Figure 18:**
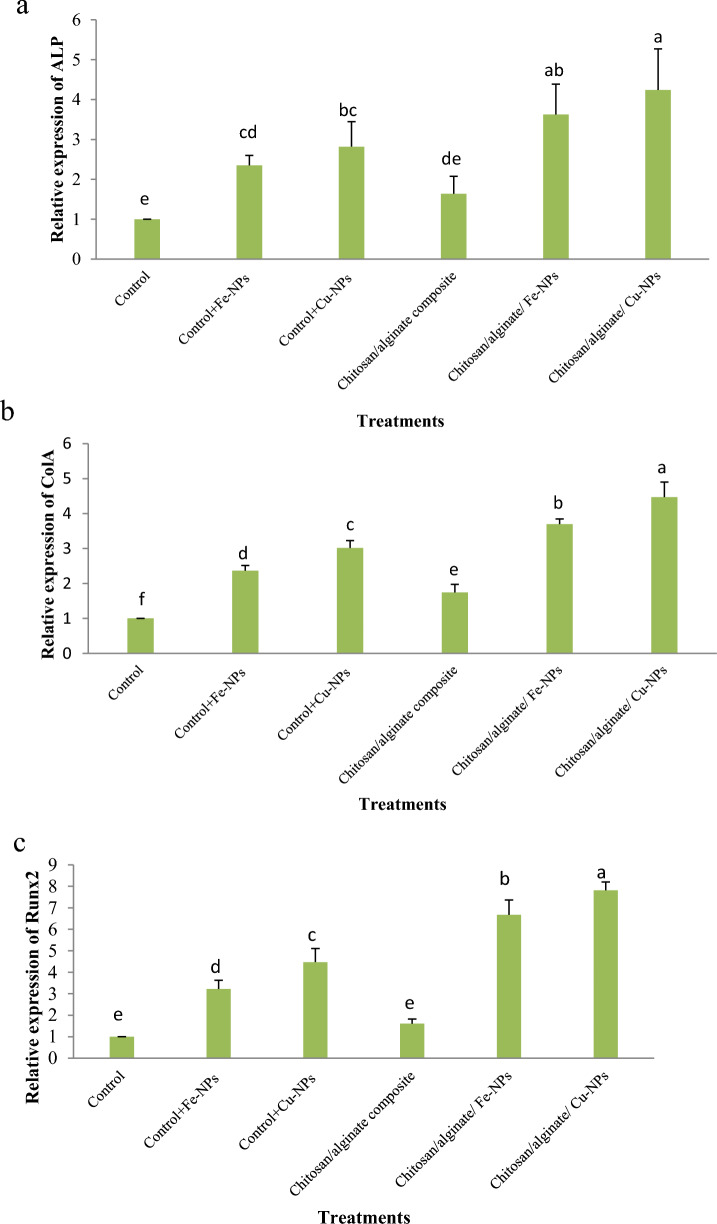
Expression analysis of the osteogenic-specific genes in ovine fetal BM-MSCs after 21 days of osteogenic differentiation (ALP, ColA, and Runx2 by qRT-PCR). In comparison to the control group (n = 3), *p* < 0.05. Significant differences in gene expression across treatment groups are indicated by various letters on distinct columns (**a**–**f**).

The potential of ovine fetal BM-MSCs towards osteocyte cells was assessed using gene expression analysis. Gene expression analysis based on in vitro differentiation induction was used to examine inherent potential (Fig. 18). Chitosan/alginate hydrogel containing CuO-NPs significantly increased the expression of the ALP gene in comparison to control, CuO or FeO- nanoparticles, and chitosan/alginate hydrogel (Fig. 18a; *p* < 0.05). ALP gene expression did not change significantly between the chitosan/alginate hydrogel groups containing CuO-NPs and those containing FeO-NPs, albeit there was a trend for the expression to decline in the hydrogel group containing FeO-NPs (*p* > 0.05). There was also no significant difference between the groups of CuO and FeO- nanoparticles (*p* > 0.05).

When ovine fetal BM-MSCs were cultured on chitosan/alginate hydrogel containing CuO-NPs, the expression of the ColA gene was significantly higher than in the control and other treatment groups (Fig. 18b; *p* < 0.05). In contrast to the control group, CuO-NPs, FeO-NPs, and chitosan/alginate hydrogel (Fig. 18b; *p* < 0.05), the presence of FeO-NPs in chitosan/alginate increased the expression of the ColA gene. The expression of the ColA gene was significantly higher in the presence of CuO-NPs in the osteogenic culture medium compared to the control groups, FeO-NPs, and chitosan/alginate hydrogel (*p* < 0.05).

Furthermore, when ovine fetal BM-MSCs were cultured on hydrogel containing CuO-NPs, the expression of the Runx2 gene was significantly higher than in other treatment groups (Fig. 18c; *p* < 0.05). FeO-NPs significantly enhanced Runx2 gene expression in chitosan/alginate hydrogel compared to control, FeO-NPs or CuO-NPs, and chitosan/alginate hydrogel (Fig. 18c; *p* < 0.05). Additionally, the differentiation medium containing CuO-NPs significantly increased Runx2 gene expression in ovine fetal BM-MSCs compared to the chitosan/alginate hydrogel group, the FeO-NPs group, and the control group (Fig. 18c; *p* < 0.05).

## Discussion

Recently, there has been increased attention towards developing a new generation of composite scaffolds based on porous chitosan that has sustained ion release and improved physicochemical properties^69^. Biocompatible 3D porous scaffolds with tissue-specific biomechanical properties can be used as a source for adhesion, implantation, and differentiation of viable cells into different types of cells^70,71^. However, several issues with biocompatibility, bio-absorption, cell adhesion capacity, and mechanical qualities still need to be resolved before artificial platforms can be synthesized to enable tissue development^72^. Metals and metal composites are highly promising materials for bone remodeling due to their high plasticity and mechanical strength^73^. Fe is a component of many enzymes and proteins necessary for metabolic processes^74^. Copper is an essential trace element in the bone matrix, enhancing the angiogenic and osteogenic properties of bone tissue engineering scaffolds^75^.

This study used lyophilization to synthesize three different forms of hydrogels: chitosan/alginate, chitosan/alginate containing FeO-NPs, and chitosan/alginate containing CuO-NPs.

Chitosan/alginate, chitosan/alginate containing FeO-NPs, and chitosan/alginate containing CuO-NPs were all observed to have stratiform pore structures in the FE-SEM images of the cross-sectional microstructures of the polysaccharide scaffolds. This was primarily because of the temperature gradients in the freezing step of scaffold preparation, where ice crystals grew in one direction which was the same as the direction of the temperature gradient.

Therefore, a stratiform pore structure emerged after the ice crystal formation phase. Similar to this, Lu et al.^49^ observed that the three-dimensional pore structure in the chitosan/alginate-containing copper scaffold was well-distributed and well-defined. According to Qasim et al.^76^, the surface of chitosan with hydroxyapatite hydrogel underwent morphological modifications. Cell adhesion and interconnections are frequently made easier by the scaffold's porosity nature^77,78^. According to Kumari et al., the addition of copper nanoparticles significantly impacted the pore diameter of scaffolds, as observed through SEM analysis. This improvement in porosity led to better cell adhesion and proliferation^79^.

Additionally, it was observed in the current study that hydrogels containing FeO-NPs or CuO-NPs provide a surface for cell attachment and differentiation. In the current study, EDS analysis revealed the presence of carbon, oxygen, chlorine, nitrogen, calcium, and sodium elements in every synthesis scaffold as well as FeO-NPs in the chitosan/alginate/FeO-NPs hydrogel and CuO-NPs in the authorized chitosan/alginate/CuO-NPs hydrogel. Similar to the current study, Sharmila et al.^80^ used EDS analysis to identify several chemicals in synthetic hydrogels. Furthermore, EDS elemental analysis has verified that berberine and Ag-NPs had successfully loaded on calcium phosphate ceramics scaffolds through silk fibroin^81^.

The mechanical test data point to the enhanced mechanical strength of FeO-NPs substituted chitosan/alginate scaffolds. During both in vitro and in vivo cell proliferation, scaffolds for bone tissue engineering must preserve adequate integrity and have sufficient mechanical strength to allow bone tissue regeneration at the site of implantation^82–84^. The trade-off between sufficient material porosity and mechanical strength is the main challenge in synthesizing polymer-based scaffolds for tissue engineering^84^. Process variables such as the pre-freezing temperature, cooling rate, polymer concentration, and composition may be used to regulate both mechanical properties and pore size^85,86^. Shi et al. created a hyaluronic acid hydrogel by combining bisphosphonate-modified HA solution and Fe3O4 nanoparticles to enhance its mechanical strength, making it more biocompatible in vivo^87^.

Contreras-Montoya et al. have synthesized novel hybrid hydrogels using Fmoc-diphenylalanine–FeNPs. The hydrogels have anisotropic properties with improved mechanical strength and water diffusion behavior^88^.

Lu et al.^49^ demonstrated that the concentration of Cu^2+^ ions released from CMC/Alg/Cu was optimal when prepared with a particular concentration of Cu nanoparticles. Additionally, they found that the CMC/Alg/Cu scaffolds, prepared in this manner, exhibited excellent biocompatibility. There were no significant cytotoxic effects on MC3T3-E1 cells and no hemolysis to human red blood cells. Our findings are consistent with their study, particularly regarding the release of FeO-NPs or CuO-NPs.

Scaffolds' structural stability and swelling characteristics are crucial for their practical application in tissue engineering. Chitosan is one of the natural polymers that swell easily in biological fluids. Studies on in vitro cultures showed that the initial swelling is preferable and that the ensuing increase in pore size enhances three-dimensional cell adhesion and proliferation^89^. However, persistent swelling can lead to a loss of mechanical integrity and generate compressive stress in the surrounding tissue.

The hydrogel's capacity to absorb water may be determined by how much it swells. The swelling ratio of the produced hydrogels in PBS is used to calculate the amount of water that is absorbed. At the end of each incubation period, the hydrogel containing FeO-NPs or CuO-NPs had a lower percentage of water absorption than the chitosan/alginate hydrogels. Water absorption decreased in the research by Zhang et al.^90^ when ZnO-NPs were present in sodium alginate-chitosan oligosaccharide hydrogel as compared to the group without nanoparticles, but the difference was not statistically significant. In their research, the hydrogel composite offered adequate blood, 3T3, and 293T cell biocompatibility as well as enough antibacterial characteristics^90^.

Lu et al.^49^ found that CMC/Alg/Cu scaffolds decreased in swelling ratio at the end of each incubation period, compared to CMC/Alg scaffolds. In line with our study, Wang et al.^91^ demonstrated that the addition of copper acetate to the alginate/carboxymethyl chitosan hydrogel resulted in a reduction in swelling compared to the hydrogel without copper acetate. The degree of reduction in swelling increased with the concentration of copper acetate.

The carboxymethyl cellulose/alginate/Spinacia oleracea extract scaffolds used by Sharmila et al.^80^ showed a high swelling capacity due to their enhanced porosity and lyophilized state. Song et al.^92^ investigated the swelling characteristics of freshly made spongy-like porous hydrogel scaffolds based on human-like collagen and discovered higher water absorption in hydrogels.

When designing a scaffold, it is important to consider its degradation rate. Ideally, the scaffold should degrade in a way that allows for new tissue regeneration. The degradation rate in the current investigation showed a substantial variation over seven days. The carboxymethyl cellulose/alginate scaffold containing spinach extract in the study by Sharmila et al.^80^ was significantly more degraded than other scaffolds. Tao et al.^93^ studied the nature of gelatin/carboxymethyl chitosan/laponite composite degradation and found that the degradation rate of the composite scaffold lowered with the addition of laponite. The degradation nature of the scaffold may depend on the reduction of phosphate-buffered lysozyme to the sugar ends, which relies on the nature of the components in the scaffold matrix^94^.

In tissue engineering, the hydrogel matrix can be used as a carrier for the direct injection of cells into a host organism without significantly reducing cell viability or function^95^. In addition, the hydrogel matrix can act as a barrier to protect the cells from the host organism's immune attack. In the present study, the proliferation of mesenchymal stem cells decreased in all the treatment groups (CuO-NPs and FeO-NPs, chitosan/alginate hydrogel with and without CuO-NPs or FeO-NPs) after 24 h when compared to the control group. However, after 72 h, the trend of cell viability among all the treatment groups was not significant, indicating that the hydrogels had good biocompatibility. Lu et al.^49^ found that the proliferation of MC3T3-E1 cells on chitosan/alginate hydrogel containing copper had no significant effect. The findings of our study were in agreement with those reported by Lu et al.^49^. Carboxymethyl chitosan/mmt and carboxymethyl cellulose/alginate scaffolds containing Spinacia oleracea extract were successful in preserving cell viability in similar research^80,96^. In our investigation, hydrogels displayed good proliferation and low cytotoxicity, as indicated by live-dead (acridine orange/ethidium bromide staining) and cytotoxicity assays.

CuO-NPs enhanced the differentiation of ovine fetal BM-MSCs into osteocyte cells in the current study. This was confirmed by various analyses, including alizarin red staining, alkaline phosphatase level, calcium deposition, and the expression of osteogenic genes. Although the exact mechanism by which CuO-NPs affect bone growth and development has not been established, it is known that copper shortage can cause anomalies in the bone^97^. In humans and animals, mild copper deficiency causes well-known bone abnormalities including osteoporosis-like lesions and bone fractures. Significant copper deficiency has been observed in osteoporotic patients compared to healthy individuals. Copper supplementation protects postmenopausal women from developing spinal bone deterioration^98,99^. The effects of copper shortage on the function of copper-dependent enzymes were seen in Menke's and occipital horn syndrome^100^.

Researchers have studied the effect of Cu^2+^ ions on mesenchymal stem cells (MSCs) or preosteoblast proliferation and osteogenic differentiation. Fromigué et al. found that Cu^2+^ ions have a distinct impact on the proliferation and differentiation processes of MSCs^101^. Rodriguez et al. observed that cell proliferation was reduced, but differentiation was increased when human MSCs were cultured in a Cu-supplemented media^42^. It was also found that Cu-containing bioactive glass scaffolds may increase osteogenic differentiation of human bone marrow stromal cells by upregulating the expression of osteogenic genes such as ALP, OCN, and osteopontin (OPN)^102^. Copper-bearing stainless steel has been shown to stimulate ALP activity, osteogenic gene expression, and new bone formation around implants in vitro and in vivo^103^.

Lu et al. ^49^ conducted a study that found that when MC3T3-E1 cells were cultured on chitosan/alginate hydrogel that contained copper, it resulted in greater ALP activity. This suggests that Cu^+2^ ions can promote osteoblast development. During the early stages of osteoblast differentiation, there is an increase in ALP activity. However, as the cells develop into a more mature phenotype and advanced matrix mineralization, there may be a subsequent reduction in ALP^104,105^.

On day 21, the alizarin red S staining showed that the presence of CuO-NPs contributed to the enhancement of calcium deposition and bone matrix formation. Monitoring the expression of genes that are relevant to osteogenesis allowed us to study the differentiation and mineralization of mesenchymal stem cells in more detail. Our research showed that the chitosan/alginate/CuO-NPs hydrogels had a significantly higher level of osteogenic-specific genes expression than the other treatment groups, after 21 days of culture. COL-I is a significant protein that forms the bone matrix and is vital for its development and mineralization.

The proliferation rate of mesenchymal stem cells isolated from human adipose tissue was maintained by the polylactic acid scaffold containing zinc, copper, and imidazole. The osteogenic differentiation has been significantly improved by this scaffold. The osteogenic differentiation activity of cells cultivated on this scaffold was enhanced by the production of alkaline phosphatase enzyme and calcium deposition^106^.

ALP activity increased with increasing Cu in the scaffold at each incubation period. These findings demonstrated that Cu in the scaffold is safe for human BM-MSCs at the doses utilized in the study and that borate bioactive glass (BG)-3Cu scaffolds had the highest ability to facilitate osteogenic differentiation of human BM-MSCs^107^. According to Wu et al.^102^ and Rodriguez et al.^42^, copper ions promote the differentiation of mesenchymal stem cells into osteoblastic cells. Previous studies have shown that a significant release of copper ions can be harmful to cells because it generates a lot of hydroxyl radicals^108^.

Another study by Zhang et al.^109^ looked at the primary adhesion activity of rat BM-MSCs on graphene oxide-copper nanocomposite covered with calcium phosphate, around 20 h after cell growth on the scaffolds showed elevated production of the adhesion-related protein, integrin beta-1. This scaffold was shown to be biocompatible and non-toxic to the cells being studied. Balanced copper concentrations imply biological activity. Based on this, they can promote bone formation in vitro, which raises ALP activity.

Early osteogenesis requires alkaline phosphatase activity^110,111^. In agreement with Wang et al.^112^ findings that the concentration of this enzyme was enhanced, a rise in alkaline phosphatase activity was seen in the group of FeO-NPs and the group of hydrogel-containing FeO-NPs in the current investigation. In addition, at a concentration of 100µg/ml in their investigation, as in the current study, no reduction in proliferation was seen. Additionally, they found that adding FeO-NPs to the culture media of human mesenchymal stem cells caused an increase in the expression of osteogenic genes and an increase in the expression of genes involved in the mitogen-activated protein kinase (MAPK) pathway. It appears that the existence of FeO-NPs or their presence in the hydrogel, which may be related to the enhanced expression of genes and proteins of this pathway, is one of the causes of the increased osteogenic differentiation in the current study. However, the expression of the genes under investigation was not examined in this study.

This specific transcription factor is regulated by the MAPK pathway and can enhance the expression of BMP-2. In earlier studies, it was found that the expression of Runx2 also increased. Runx2 is a necessary gene for establishing an osteogenic lineage from mesenchymal stem cells^113–116^. When there is an increased expression of Runx2, it leads to the overexpression of osteogenic genes such as ALP, collagenase, and osteocalcin, among others^117,118^. Consequently, the MSCs differentiate into osteoblasts. Recent research has revealed that FeO-NPs can promote the proliferation of human BM-MSCs by eliminating intracellular H_2_O_2_^119^. Further studies are required to determine the best conditions for in vitro and in vivo differentiation of the human MSCs into osteocyte cells seeded on the current scaffold.

## Conclusion

To provide the best conditions for the proliferation and osteogenic differentiation of ovine fetal BM-MSCs, three sets of hydrogel scaffolds employing chitosan, alginate, CuO-NPs, or FeO-NPs were created in this study. Because of the hydrogel groups' evenly and abundantly linked porosity structure, which has produced a theme for cell adhesion, there was no discernible difference in the decrease of proliferation after 72 h between treatment groups. However, hydrogels containing nanoparticles, particularly those containing CuO-NPs, showed the greatest osteogenic differentiation of ovine fetal BM-MSCs.

### Supplementary Information

Supplementary Figure 1.

Supplementary Table 1.

## Data Availability

The data that support the findings of this study are available from the corresponding upon reasonable request.
