# Author Correction: Comparison of the differentiation of ovine fetal bone-marrow mesenchymal stem cells towards osteocytes on chitosan/alginate/CuO-NPs and chitosan/alginate/FeO-NPs scaffolds

**DOI:** 10.1038/s41598-024-59544-z

**Published:** 2024-04-16

**Authors:** Leila Soltani, Kambiz Varmira, Maryam Nazari

**Affiliations:** 1 Department of Animal Sciences, College of Agriculture and Natural Resources, Razi University, Kermanshah, 67144-14971 Iran; 2 Research Center of Oils and Fats, Kermanshah University of Medical Sciences, Kermanshah, Iran; 3 Applied Chemistry Department, Faculty of Chemistry, Razi University, Kermanshah, Iran

Correction to: *Scientific Reports* 10.1038/s41598-023-50664-6, published online 02 January 2024

In the original version of this Article, Kambiz Varmira was omitted as a co-corresponding author. Correspondence and requests for materials should also be addressed to varmira@yahoo.com.

The original Article has been corrected.

