# Supplementary figures and images for: Comparison of the differentiation of ovine fetal bone-marrow mesenchymal stem cells towards osteocytes on chitosan/alginate/CuO-NPs and chitosan/alginate/FeO-NPs scaffolds
Source: Sci Rep. 2024 Jan 2;14:161. doi: 10.1038/s41598-023-50664-6 (PMC10762099; doi:10.1038/s41598-023-50664-6)

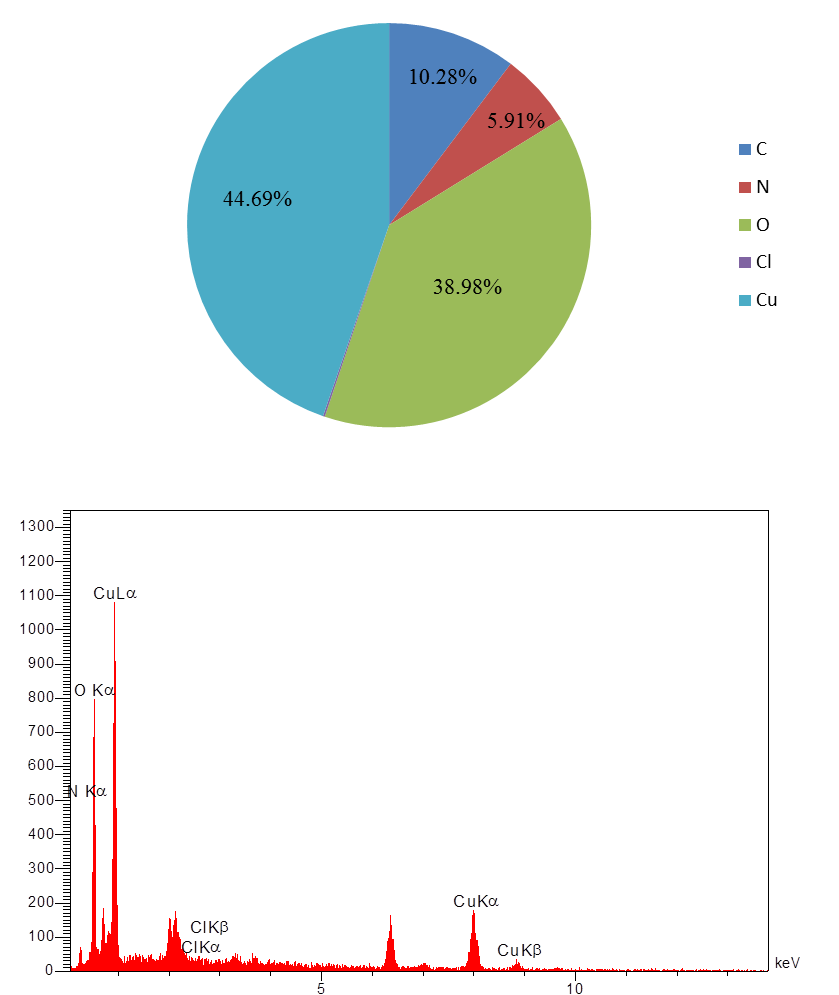


a)


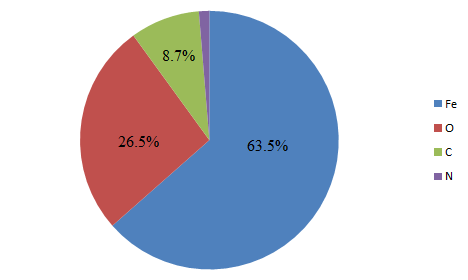


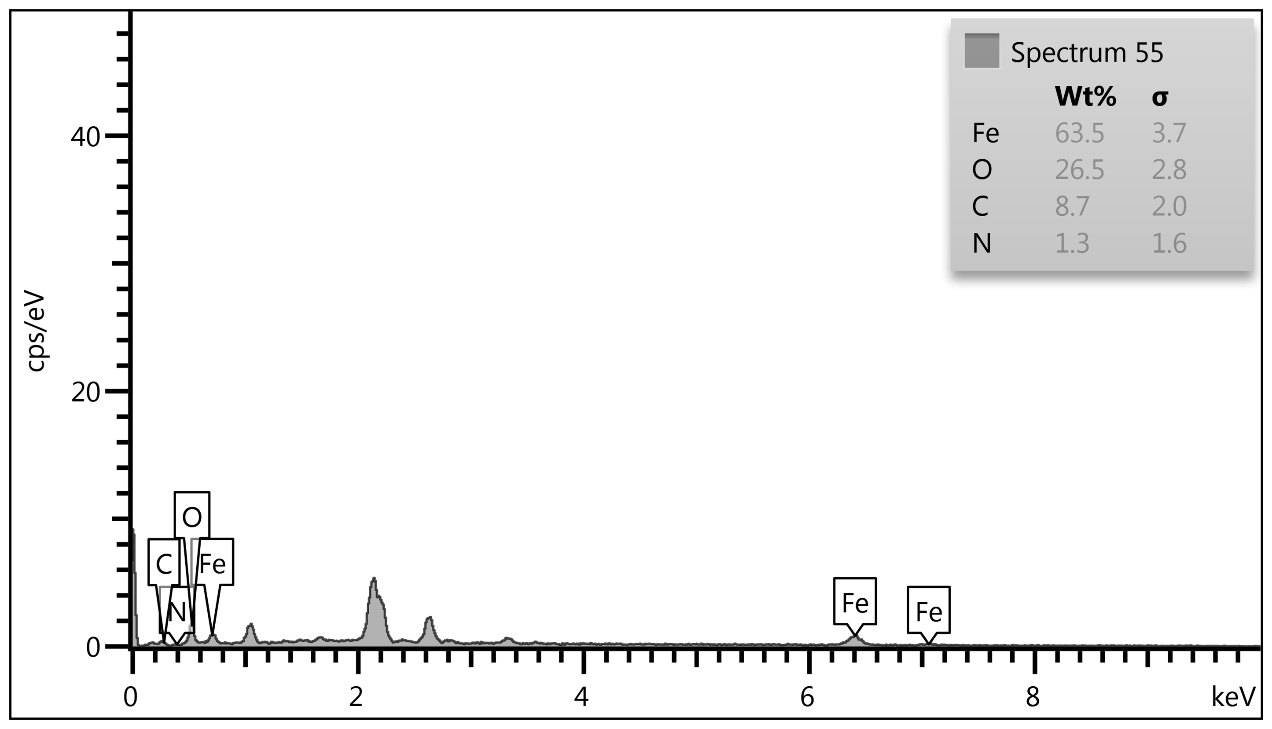


b)

Supplementary Figure 1: EDS of a) CuO-NPs and b) FeO-NPs

Supplement: Supplementary file 1 — Supplementary Figure 1. [file 41598_2023_50664_MOESM1_ESM.docx]
