# Supplementary material for: Comparison of the differentiation of ovine fetal bone-marrow mesenchymal stem cells towards osteocytes on chitosan/alginate/CuO-NPs and chitosan/alginate/FeO-NPs scaffolds
Source: Sci Rep. 2024 Jan 2;14:161. doi: 10.1038/s41598-023-50664-6 (PMC10762099; doi:10.1038/s41598-023-50664-6)
Supplement: Supplementary file 2 — Supplementary Table 1. [file 41598_2023_50664_MOESM2_ESM.docx]

[Supplementary Table 1](https://static-content.springer.com/esm/art%3A10.1038%2Fs41598-023-43441-y/MediaObjects/41598_2023_43441_MOESM3_ESM.docx): FTIR frequency range and functional groups

| Component | Frequency | Characterization |
| --- | --- | --- |
| alginate | 3454 cm^-1^ | -OH group from carboxyl |
| alginate | 1622 and1411 cm^-1^ | asymmetric and symmetric vibration of -COOH |
| alginate | 1021 cm^-1^ | C-O vibration from carboxylic acid |
| Chitosan | 1615 cm^-1^ | N-H |
| Chitosan | 3434 cm^-1^ | N-H (overlapping of O-H and NH_2_ vibration) |
| Chitosan | 1081 cm^-1^ | C-N stretching vibration |
| Chitosan | 1329 – 1426 cm^-1^ | deformation from C-OH vibration |
| alginate-chitosan | 3420 cm^-1^ | -OH and NH_2_ vibration |
| alginate-chitosan | 1622 cm^-1^ | N-H |
| alginate-chitosan | 1622 and 1411 cm^-1^ | asymmetric and symmetric vibration of -COOH |
| alginate-chitosan-CuNPs | 1620 and 1386 cm^-1^ | asymmetric and symmetric vibration of -COOH |
| alginate-chitosan-CuNPs | 620 and 471 cm^-1^ | Cu-O |
| alginate-chitosan-CuNPs | 3414 cm^-1^ | -OH and NH_2_ vibration |
| alginate-chitosan-FeO NPs | 1620 and 1402 cm^-1^ | asymmetric and symmetric vibration of -COOH |
| alginate-chitosan-FeO NPs | 621 cm^-1^ | Fe-O |
| alginate-chitosan-FeO NPs | 3414 cm^-1^ | -OH and NH_2_ vibration |
| CuNPs | 611 and 481 cm^-1^ | Cu-O |
| FeO NPs | 601 cm^-1^ | Fe-O |
